# Magnetic and Magnetocaloric Properties of the A_2_LnSbO_6_ Lanthanide Oxides on the Frustrated *fcc* Lattice

**DOI:** 10.1021/acs.inorgchem.3c01137

**Published:** 2023-06-16

**Authors:** EliseAnne C. Koskelo, Nicola D. Kelly, Liam A. V. Nagle-Cocco, Joshua D. Bocarsly, Paromita Mukherjee, Cheng Liu, Qiang Zhang, Siân E. Dutton

**Affiliations:** † Department of Physics, University of Cambridge, Cambridge CB3 0HE, United Kingdom; ‡ Yusuf Hamied Department of Chemistry, University of Cambridge, Cambridge CB2 1EW, United Kingdom; ¶ Neutron Scattering Division, Oak Ridge National Laboratory, Oak Ridge, Tennessee 37830, United States

## Abstract

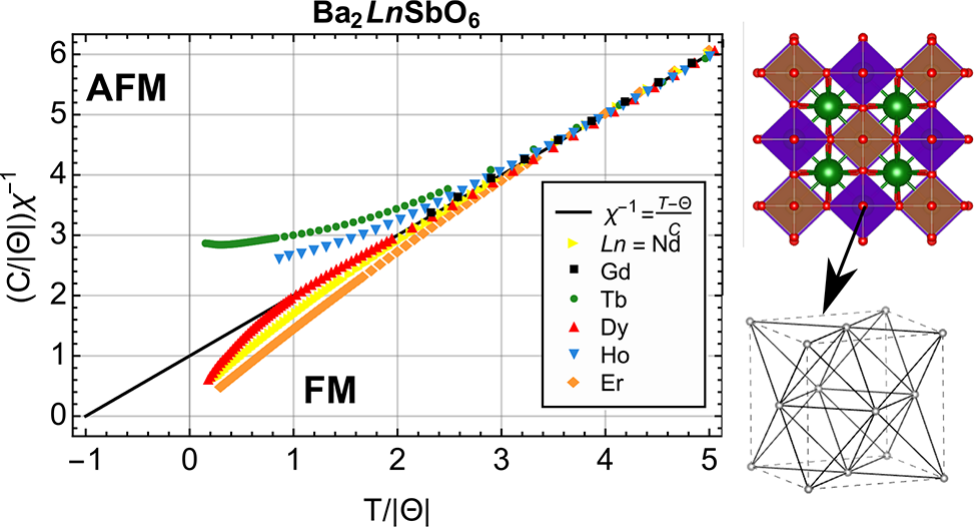

Frustrated lanthanide oxides are promising candidates for cryogen-free magnetic refrigeration due to their suppressed ordering temperatures and high magnetic moments. While much attention has been paid to the garnet and pyrochlore lattices, the magnetocaloric effect in frustrated face-centered cubic (*fcc*) lattices remains relatively unexplored. We previously showed that the frustrated *fcc* double perovskite Ba_2_GdSbO_6_ is a top-performing magnetocaloric material (per mol Gd) because of its small nearest-neighbor interaction between spins. Here we investigate different tuning parameters to maximize the magnetocaloric effect in the family of *fcc* lanthanide oxides, A_2_LnSbO_6_ (A = {Ba^2+^, Sr^2+^} and Ln = {Nd^3+^, Tb^3+^, Gd^3+^, Ho^3+^, Dy^3+^, Er^3+^}), including chemical pressure via the A site cation and the magnetic ground state via the lanthanide ion. Bulk magnetic measurements indicate a possible trend between magnetic short-range fluctuations and the field-temperature phase space of the magnetocaloric effect, determined by whether an ion is a Kramers or a non-Kramers ion. We report for the first time on the synthesis and magnetic characterization of the Ca_2_LnSbO_6_ series with tunable site disorder that can be used to control the deviations from Curie–Weiss behavior. Taken together, these results suggest *fcc* lanthanide oxides as tunable systems for magnetocaloric design.

## Abstract 2

We investigate different tuning parameters to maximize the magnetocaloric effect in *fcc* lanthanide oxides, A_2_LnSbO_6_ (A = {Ba^2+^, Sr^2+^} and Ln = {Nd^3+^, Tb^3+^, Gd^3+^, Ho^3+^, Dy^3+^, Er^3+^}), including chemical pressure via the A site cation and the magnetic ground state via the lanthanide ion. Bulk magnetic measurements indicate a possible trend between magnetic short-range fluctuations and the field-temperature phase space of the magnetocaloric effect, determined by whether an ion is a Kramers or a non-Kramers ion. The Ca_2_LnSbO_6_ series has tunable site disorder that can be used to control the deviations from Curie−Weiss behavior.

## Introduction

Cooling is a vital part of modern technology from satellite sensors to medical imaging to quantum computing. While cooling to *T* ≈ 2 K and *T* ≪ 2 K can be achieved using liquid He and 3-He, respectively, He is a depleting resource, and the search for alternatives based on magnetocaloric, mechanocaloric, and elastocaloric materials is an ongoing area of research.^1−4^ Frustrated magnets have been identified as promising candidates for cryogen-free magnetic refrigeration due to their suppressed ordering temperatures and degenerate ground states.^5^ Cooling is accomplished via the magnetocaloric effect (MCE) in which an applied magnetic field is used initially to order paramagnetic spins; once the field is removed, spins become disordered and the resulting magnetic entropy change is used to absorb heat. For a given change in field, Δ*H*, the adiabatic temperature change (Δ*T*)_*S*_ of a material is proportional to its change in magnetic entropy at constant temperature Δ*S*_m_.^6^ The lowest possible temperature is limited by the long-range magnetic ordering temperature, with reports of an enhanced MCE just above the magnetic ordering temperature in GdPO_4_^7^ and Gd(OH)F_2_.^8^

Frustrated lanthanide oxides are well suited to magnetic refrigeration due to their large total angular momentum (high magnetic entropy) and high chemical stability compared to paramagnetic salts in traditional adiabatic refrigeration.^9^ Due to the localized nature of the 4f electrons, the magnetic properties are highly dependent on competing superexchange, dipolar, and crystal electric field (CEF) interactions. For example, in the Ln_3_Mg_2_Sb_3_O_14_ kagome family, Dy_3_Mg_2_Sb_3_O_14_ is an Ising magnet that exhibits an emergent charge-ordered state at ∼0.3 K;^10^ Pr_3_Mg_2_Sb_3_O_14_ is a van Vleck paramagnet; and Tb_3_Mg_2_Sb_3_O_14_ and Ho_3_Mg_2_Sb_3_O_14_ each have a ground state that depends on the CEF, superexchange, and dipolar interactions.^11^ While the magnetocaloric effect has been studied in a variety of frustrating lattices, including the Shastry–Sutherland latttice (e.g., Ln_2_Be_2_GeO_7_),^12^ quasi-1D chains (e.g., Ca_4_LnO(BO_3_)_3_),^13^ garnets (e.g., Ln_3_A_2_X_3_O_12_),^14^ and pyrochlores (e.g., Ln_2_B_2_O_7_, B = {Ti,Sn}),^15^ the family of face-centered cubic (*fcc*) lanthanide oxides remains relatively unexplored. It is therefore important to obtain a holistic study of the magnetocaloric effect of the Ln *fcc* family in connection to its magnetic properties.

**Figure 1:**
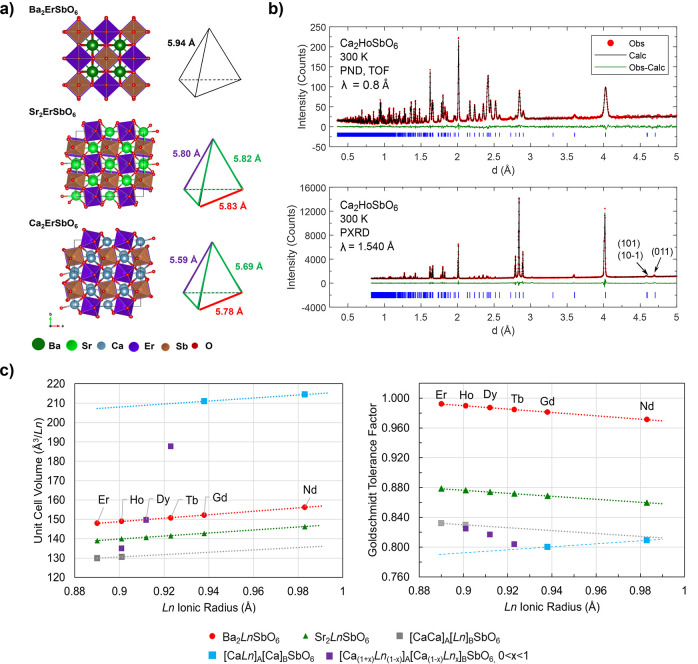
(a) Crystal structures of A_2_ErSbO_6_ where A = Ba^2+^ (*Fm*3̅*m*), A = Sr^2+^ (*P*2_1_/*n*), and A = Ca^2+^ (*P*2_1_/*n*). All Ln^3+^ ions are distributed on an *fcc* lattice, which is composed of edge-sharing tetrahedra. The side-lengths of the tetrahedron for each compound, shown in the right part of the subfigure, are uniform for the cubic Ba compound and distorted for the monoclinic Sr and Ca compounds. The smaller ionic radius of Ca compared to Sr results in increased distortion, with side-length differences of ∼3.5% compared to 0.5%. (b) Combined Rietveld refinement of room-temperature PND (top panel) and PXRD data (bottom panel) for Ca_2_HoSbO_6_. The two peaks at *d* ≈ 4.63 and 4.76 Å, corresponding to the reflections (101)/(10 1̅) and (011), are much more suppressed in Ca_2_HoSbO_6_ compared to Ca_2_NdSbO_6_, indicating only a small amount (∼8.3(2)% of all Ho) of Ln occupancy on the A site. (c) Volume of the unit cell and Goldschmidt tolerance factor of A_2_LnSbO_6_, where A = {Ba, Sr, Ca}, as a function of the Ln ionic radius normalized by the number of Ln ions per unit cell. Points correspond to the measured structural data, and the lines correspond to lines of best fit.

Systems of frustrated *fcc* magnetic sublattices can be formed using a double perovskite structure, A_2_BB′O_6_, in which the rock-salt ordering of the magnetic B and nonmagnetic B′ cations forms two networks of B–B and B′–B′ edge-sharing tetrahedra,^16^Figure 1a). The A site cation lies in the cavity between the BO_6_ and B′O_6_ octahedra and can result in either a cubic *Fm*3̅*m* structure when the ionic radius is larger (e.g., for Ba^2+^) and the *fcc* lattice is undistorted or the monoclinic *P*2_1_/*n* structure when the ionic radius is smaller (Sr^2+^ or Ca^2+^)^16^ and the reduction in symmetry results in a distorted *fcc* lattice. Nearest-neighbor superexchange between magnetic ions (*J*_1_) occurs along the Ln–O–O–Ln pathways, while next-nearest-neighbor (*nnn*) superexchange (*J*_2_) occurs through the Ln–O–Sb–O–Ln routes.^17^

The magnetic ground states across the *fcc* family of double perovskites are exceedingly diverse depending on the B and B′ site cations. For example, the 3d transition metals Ni^2+^, high spin Mn^2+^, Ru^5+^, Co^3+^, and Os^7+^ lead to ordered antiferromagnets at ∼10–50 K,^18−22^ while Fe^3+^ and Re^5+^/Re^6+^ lead to spin glass states at ∼15–30 K. Notably, these spin glass effects are shown to persist when chemical pressure is tuned via the A site cation (e.g., Ba^2+^ to Ca^2+^), while the antiferromagnetic ordering temperature can be further suppressed in Co-based double perovskites for larger A site cations.^20,23^ The B site cation Mo^5+^ is reported to exhibit an exotic valence bond glass state,^17,24^ and the rare-earth iridates Ba_2_LnIrO_6_ all remain paramagnetic down to 2 K except for Pr^3+^.^25^

Our previous work showed that, for Heisenberg spin systems, the magnetocaloric effect (per ion) is enhanced when the system is frustrated, but its superexchange is minimal.^26^ For example, the frustrated *fcc* Ba_2_GdSbO_6_ has a nearest-neighbor (*nn*) superexchange *J*_1_ of 10 mK and does not order down to 400 mK. *fcc* Ba_2_GdSbO_6_ attains a magnetic entropy change Δ*S*_m_ of −15.8(1) J/K/mol_Gd_ compared to Gd_3_Ga_5_O_12_ with *J*_1_ = 100 mK and an entropy of −13.0 J/K/mol_Gd_ at 2 K and 7 T. The related compound Sr_2_GdNbO_6_ with a lower symmetry and a distorted *fcc* lattice is similarly high performing, attaining −15.5 J/K/mol_Gd_ near its ordering temperature of 3 K and 7 T, but orders at 3 K, highlighting that the d^0^ versus d^10^ electronic configuration of the nonmagnetic *B* site can play a role in the magnetic properties of *fcc* lanthanide oxides.^26,27^

The *fcc* lanthanide oxides, A_2_LnBO_6_ (A = {Ba^2+^, Sr^2+^}), are promising magnetocaloric materials because prior reports have shown a lack of ordering to 2 K and minimal correlations between spins or van Vleck paramagnetism. Karunadasa et al. studied A_2_LnSbO_6_, A = {Ba^2+^, Sr^2+^} and Ln = {Gd^3+^, Dy^3+^, Ho^3+^}, a subset of the materials studied in this work, and reported negative Curie–Weiss temperatures and postulated they were possible spin liquid candidates.^16^ Subsequent low-temperature neutron diffraction measurements, however, showed an absence of short-range correlations between Ln ions to 70 mK in the *fcc* Ba_2_HoSbO_6_ and Ba_2_ErSbO_6_. Both compounds have been shown to be well described by van Vleck paramagnetism due to low-lying excited states.^28,29^

In this report, we investigate the A_2_LnSbO_6_ (A = {Ba^2+^, Sr^2+^} and Ln = {Nd^3+^, Tb^3+^, Gd^3+^, Ho^3+^, Dy^3+^, Er^3+^}) family to explore effects of lattice symmetry (tuned via chemical pressure) as well as single-ion anisotropy and the magnetic ground state (via a magnetic cation) on the magnetocaloric effect. We synthesize a new series of *fcc* materials, Ca_2_LnSbO_6_, and find that site-disorder can be tuned with slow cooling during synthesis. We find a trend between the deviations in the magnetic susceptibility of a material from Curie–Weiss behavior and the field-temperature phase space at which its magnetocaloric effect is maximized. Furthermore, we show that these magnetic properties are determined by whether the magnetic cations are Kramers versus non-Kramers ions. Taken together, these results indicate that the A_2_LnSbO_6_ family of *fcc* magnets is a tunable system for magnetic refrigeration.

## Experimental Methods

### Solid-State Synthesis

Polycrystalline samples of A_2_LnSbO_6_ (A = {Ba^2+^, Sr^2+^, Ca^2+^} and Ln = {Nd^3+^, Tb^3+^, Gd^3+^, Ho^3+^, Dy^3+^, Er^3+^}) were synthesized by mixing stoichiometric amounts of the reactant powders antimony(V) oxide (99.9998%, Alfa Aesar Puratronic), lanthanide (III) oxide (99.999%, Alfa Aesar REacton) for all Ln except Tb, and the appropriate alkaline earth carbonate, namely, barium carbonate (99.997%, Alfa Aesar Puratronic), strontium carbonate (99.99%, Alfa Aesar), or calcium carbonate (99.99%, Alfa Aesar Puratronic). The lanthanide precursor for all Tb compounds was terbium (III,IV) oxide (99.9%, Alfa Aesar REacton). The precursors Nd_2_O_3_, Tb_4_O_7_, Gd_2_O_3_, and Dy_2_O_3_ were dried in air at 800 °C for 12 h prior to weighing. Reactants were ground using a mortar and pestle and heated in air at 1400 °C for 24 h for 2–4 cycles. Intermittent grindings between heatings were conducted. The Ba and Sr series were cooled to room temperature using furnace cooling, while the effect of “slow cooling” (1 °C/min) was explored to eliminate site disorder in the Ca series.

### Structural Characterization

Room-temperature powder X-ray diffraction (XRD) measurements were conducted using a Bruker D8 Advance diffractometer (Cu Kα radiation, λ = 1.54 Å). Data were taken with a resolution of Δ(2θ) = 0.01° from 2θ = 15° to 2θ = 150° for an overall collection time of 2–3 h. To minimize preferred orientation effects, the sample stage was rotated at 30 rpm during data collection. Additional high-resolution powder diffraction measurements were conducted for the Ca_2_LnSbO_6_ samples at the I11 beamline at the Diamond Light Source using a position-sensitive detector at 100 K. Data were collected with λ = 0.826866 Å from 2θ = 8° to 2θ = 100°, with an overall collection time of 1 min. The powder sample was mounted in a 0.28 mm diameter borosilicate capillary. For Ca_2_HoSbO_6_, Ca_2_NdSbO_6_, and Ca_2_ErSbO_6_, high-resolution neutron powder diffraction measurements were conducted at room temperature using the POWGEN diffractometer at the Spallation Neutron Source (bank 1, 0.27 < λ < 1.33 Å with a center wavelength of 0.8 Å). Each (∼1 g) powder sample was mounted in a 6 mm diameter vanadium can, and data were collected to cover *d* = 0.1340–8.200 Å with 0.001 < *Δd*/*d* < 0.008 and an overall collection time of 2.5–3.25 h.

Powder X-ray diffraction (XRD) data were refined using the Rietveld method^30^ in the Diffrac.Suite TOPAS5 program.^31^ A pseudo-Voigt function was used to model peak shape,^32^ and the background was fit using a 13-term Chebyshev polynomial. Except for Ca_2_LnSbO_6_ (Ln = {Nd, Gd, Tb, Dy, Ho, Er}), where synchrotron XRD data or powder neutron diffraction data were available, all Debye–Waller factors were set to the literature-reported values from powder neutron diffraction.^16,33^ Where available, joint Rietveld refinements were conducted on the powder neutron and powder X-ray diffraction data together. A cylindrical correction was used to correct for capillary absorption in the I11 data as well as a Lorentzian/Gaussian model to account for strain broadening effects on the peak shape.^34^

### Bulk Magnetic Measurements

Magnetic susceptibility χ(*T*) = d*M*/d*H* and isothermal magnetization *M*(*H*) measurements were conducted using a Quantum Design Magnetic Properties Measurement System (MPMS) with a superconducting interference device (SQUID) and Physical Properties Measurement System (PPMS). Susceptibility measurements were made at μ_0_*H* = 0.01 T in the low-field limit where χ(*T*) = d*M*/d*H* ≈ *M*/*H* in zero-field-cooled conditions from 1.8 to 300 K. The *M*(*H*) measurements are linear at this field, confirming that this linear approximation of χ is valid. *M*(*H*) measurements were made at temperatures of 2 to 20 K in steps of 2 K over a field range of 0 to 7 T in steps of 0.2 T. For A_2_LnSbO_6_ (A = {Ba, Sr} and Ln = {Dy, Ho}) and Sr_2_GdSbO_6_, the *M*(*H*) data from 10 to 20 K were taken in steps of 5 K.

Further details on the analysis of the magnetic measurements and calculations on the magnetocaloric effect can be found in the Supporting Information.

## Experimental Results

All compounds formed with the nominal Ln^3+^ oxidation state. The following compounds have also been found to be stable but are not included in this report: Ba_2_YbSbO_6_,^35^ Sr_2_YbSbO_6_,^36^ and Ba_2_PrSbO_6_,^37^.

### Structural Characterization

#### Ordered *fcc* Magnetic Sublattices: A = {Ba, Sr}

The X-ray diffraction of all A = Ba^2+^ compounds are well fit to the cubic *Fm*3̅*m* space group.^16,28,29^ The Ln^3+^ and Sb^5+^ ions order in a rock-salt arrangement on octahedrally coordinated B sites with no observed B/B′ antisite disorder. Ba^2+^ cations lie at cavities formed by the BO_6_ octahedra, forming a double perovskite structure, Figure 1a). As in ref (38), Ba_2_NdSbO_6_ was refined in the *R*3̅ group, which arises due to rotations of the Ln-O and Sb–O octahedra about the [111] axis.

The A = Sr^2+^ series adopts the related monoclinic *P*2_1_/*n* space group, where the fully ordered Ln–O and Sb–O octahedra undergo rotations about [011]_cubic_ and [100]_cubic_ (*a*^–^*b*^–^*b*^–^ in Glazer’s notation, Figure 1a).^16^ The lower symmetry of the A = Sr^2+^ crystal results in the reduction of coordination number of the A site from 12 to 8 and three independent O positions. Tables S2–S4 detail the crystal structure parameters for A_2_LnSbO_6_ (Ln = {Nd–Er} and A = {Ba, Sr}). For both A = {Ba, Sr}, the rock-salt arrangement of Ln and Sb ions implies an *fcc* magnetic sublattice of edge-sharing tetrahedra.

The symmetry of the Ln–Ln tetrahedra, like the overall symmetry of the crystallographic cell, is determined by the A site cation,^16^Figure 1. In the case of A = Ba, each tetrahedron is uniform, while for A = {Sr, Ca} each tetrahedron is distorted, with ∼1.0–3.5% side-length differences (as discussed, Ca_2_LnSbO_6_ (Ln = {Er, Ho}) are the only compounds in the Ca series with an ordered *fcc* magnetic sublattice). The volume of the unit cell and size of the Ln–Ln tetrahedra, for A = {Ba, Sr} and *fcc* Ca_2_HoSbO_6_ and Ca_2_ErSbO_6_, scales linearly with the ionic radius of Ln^3+^, Figure 1c, as expected from a close-packing model of the structure.^39^

#### Tunable Site-Disordered Magnetic Sublattices: A = Ca

All A = Ca materials were refined in the *P*2_1_/*n* space group, Tables 1 and S1, consistent with prior reports for Ca_2_NdSbO_6_ and Ca_2_HoSbO_6_.^26,43,44^ The diffraction patterns contain two PXRD/PND peaks at *d* ≈ 4.6–4.8 Å, Figures 1b and S2, indicative of site disorder. Reports for A_2_BSbO_6_ (A = {Sr^2+^, Ca^2+^}, B = {La^3+^, Sm^3+^}) show that these additional peaks correspond to (011) and (101)/(−101) in the monoclinic unit cell and emerge due to A site occupancy of the Ln^3+^ ions.^45^ As in ref (45), partial occupancy of the Ln ions on the A site was refined using the formula [Ca_(1+*x*)_Ln_(1–*x*)_]_*A*_[Ca_(1–*x*)_Ln_*x*_]_*B*_SbO_6_ (full B site (A site) occupancy corresponds to *x* = 1 (*x* = 0, with 1:1 site-disorder of Ca and Ln on the A sites)). For furnace cooling, all Ca_2_LnSbO_6_ compounds except Ln = Er have some Ln ions on the A site, with Ln = {Dy, Ho} exhibiting *x* = 0.552(3) and *x* = 0.847(2), respectively, after three 1400 °C heatings. In none of the Ca-containing systems was B/B’ antisite disorder observed.

**Table 1:** Structural parameters of Ca_2_LnSbO_6_ (Ln = {Nd^3+^, Gd^3+^, Tb^3+^, Dy^3+^, Ho^3+^, Er^3+^})^a^

Ln		Nd	Gd	Tb	Dy	Ho	Er
Ca_2_LnSbO_6_							
*a* (Å)		5.62758(4)	5.58025(2)	5.57694(6)	5.58565(6)	5.59657(3)	5.59288(3)
*b* (Å)		5.86700(4)	5.84820(2)	5.83702(7)	5.82026(6)	5.80017(3)	5.78472(3)
*c* (Å)		8.11704(5)	8.07706(2)	8.06982(9)	8.06445(9)	8.05155(4)	8.03641(5)
β (deg)		89.8209(5)	90.3253(2)	90.2549(7)	90.0769(8)	90.0812(4)	90.1065(4)
4*e*: Ca_1_/Ln_2_	*x*	0.0150(1)	–0.0174(1)	–0.0057(5)	0.0179(5)	0.0141(2)	0.0129(2)
	*y*	0.0555(1)	0.05939(7)	0.0578(2)	0.0576(3)	0.0534(1)	0.0516(1)
	*z*	0.2530(1)	0.25403(8)	0.2481(3)	0.2512(6)	0.2517(2)	0.2511(2)
	occupancy Ca	0.5	0.5	0.607(1)	0.776(1)	0.959(1)	1.0
	occupancy Ln	0.5	0.5	0.393(1)	0.224(1)	0.041(1)	0.0
	B_iso_ (Å^2^)	0.497(8)	0.90(1)	0.59	0.59	0.57(1)	0.46(1)
2*d*: Ln_1_/Ca_2_	occupancy Ln	0.0	0.0	0.215(3)	0.552(3)	0.917(2)	1.0
	occupancy Ca	1.0	1.0	0.785(3)	0.448(3)	0.083(2)	0.0
	B_iso_ (Å^2^)	0.50(2)	0.68(8)	0.99	0.99	0.217(8)	0.31(1)
2*c*: Sb	B_iso_ (Å^2^)	0.18(1)	0.54(1)	0.40	0.40	0.27(1)	0.28(1)
4*e*: O(1)	*x*	0.2875(2)	0.1660(8)	0.313(2)	0.296(2)	0.2877(2)	0.2880(2)
	*y*	0.3222(2)	0.2149(7)	0.342(2)	0.335(2)	0.3166(2)	0.3152(2)
	*z*	0.0517(1)	–0.0728(6)	0.0480(2)	0.063(2)	0.0525(1)	0.0526(1)
	B_iso_ (Å^2^)	0.89(1)	1.25(5)	0.99	0.99	0.73(1)	0.63(1)
4*e*: O(2)	*x*	0.3280(2)	0.2089(8)	0.319(2)	0.328(3)	0.3221(2)	0.3200(2)
	*y*	0.2825(2)	0.1769(7)	0.290(2)	0.267(2)	0.2849(2)	0.2851(2)
	*z*	0.4320(1)	0.5511(6)	0.453(2)	0.442(2)	0.4376(1)	0.4392(2)
	B_iso_ (Å^2^)	0.75(1)	1.25(5)	0.99	0.99	0.68(1)	0.58(1)
4*e*: O(3)	*x*	0.8857(2)	1.1205(7)	0.885(2)	0.882(2)	0.8906(2)	0.8930(2)
	*y*	0.4467(2)	0.4390(7)	0.436(2)	0.450(2)	0.4530(1)	0.4553(2)
	*z*	0.2287(1)	0.2254(5)	0.216(1)	0.236(2)	0.2322(1)	0.2335(1)
	B_iso_ (Å^2^)	0.62(1)	1.25(5)	0.99	0.99	0.55(1)	0.51(1)
Ln_3_SbO_7_ (wt %)			0.48(1)	0.68(3)			
*R*_wp_		5.36	3.7	10.5	7.41	5.11	6.00
χ^2^		1.39	5.80	1.49	1.18	1.43	1.43
		PND/PXRD	SPXRD	PXRD	PXRD	PND/PXRD	PND/PXRD

a Joint Rietveld refinements of PXRD and PND data were conducted for Ln = {Nd, Ho, Er}, while only PXRD was used for Ln = {Tb, Dy}. High-resolution x-ray synchrotron measurements at 100 K were carried out at the I11 beamline for Ca_2_GdSbO_6_ in our previous study, reported here for ease of comparison.^26^ Refinements were carried out in the space group *P*2_1_/*n*, with Ca_1_/Ln_2_ on the 4*e* sites (*x*, *y*, *z*), Ln_1_/Ca_2_ on the 2*d* sites (1/2, 0, 0); Sb on the 2*c* sites (0, 1/2, 0); and O(1), O(2), and O(3), on the 4*e* sites (*x*, *y*, *z*). Er ions were restricted to the B site in the final refinement, since allowing for site-disorder refined to a fully ordered phase. In the case where a small impurity peak was present near 2θ = 29°, an impurity phase of Ln_3_SbO_7_ was fit using the reported structure.^40−42^ In the final row, PND/PXRD indicates a joint Rietveld refinement on room-temperature powder neutron diffraction data and lab X-ray diffraction data, PXRD represents a refinement on room-temperature lab X-ray diffraction data, and SPXRD represents refinement on low-temperature (100 K) synchrotron X-ray diffraction data.

To investigate the possibility of tunable site ordering, two additional heatings were conducted for Ln = {Dy, Ho}, with a slow-cooling rate of 1 °C/min. Rietveld refinements after slow-cooling showed increased B site occupancy of *x* = 0.672(3) for Dy and *x* = 1.000 for Ho. Ca_2_TbSbO_6_ was fired at 1400 °C for three 24 h periods with a 1 °C/min cooling rate but exhibited site disorder, with *x* = 0.215(3), suggesting a slower rate may be necessary. An additional synthesis of Ca_2_ErSbO_6_ carried out at a lower temperature (1300 °C) with slow-cooling exhibited some (*x* = 0.937(1)) site disorder (see Table S1), suggesting that disorder may also depend on the synthesis temperature.

Crystal structure parameters for Ca_2_LnSbO_6_ are provided in Table 1 for room temperature and Table S1 for 100 K. For the larger Ln = {Gd^3+^, Nd^3+^} ions, Ln^3+^ is found almost exclusively on the A site in Ca_2_LnSbO_6_.^43,46^ The possibility of A site ordering in Ca_2_GdSbO_6_ has been addressed in ref (26). Superstructure peaks expected of a A site ordering are not evident in the high-resolution X-ray synchrotron data.

The Goldschmidt tolerance factor *t* was calculated to analyze the origin of site-disorder in the A = Ca series, Figure 1c). *t* predicts whether the ionic radii of the A site cation and B site cation are well-scaled for a cubic structure in which A site cations lie at the cavities of B–O octahedra.^47^ It can be extended to double perovskites by computing an average B site ionic radius so that
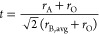
1where *r*_B,avg_ = 1/2*r*_Sb_ + 1/2*r*_Ln_.^39^ A value of *t* ≈ 1 predicts a cubic structure, while *t* > 0.7 predicts a stable perovskite structure but with tilting of the B–O/B′–O octahedra and/or B–O/B′–O bond-length distortion.^38,39^

For all *fcc* materials (Ln ions on the B site), *t* is predicted to be stable (*t* > 0.81) and scales linearly with Ln ionic radius. The calculated *t* for A = {Ba, Sr} agree with the observed structures. Conversely, for [CaLn]_A_[Ca]_B_SbO_6_, in which the Ln ions lie on the A sites, *t* ≤ 0.81. The Goldschmidt tolerance factor of materials that exhibit site mixing, namely Ca_2_LnSbO_6_ (Ln = {Tb,Dy,Ho}), lie at intermediate values.

Since *t* ≈ 1 in stable perovskite structures, these results suggest that the Ln ions should lie preferentially on the B site. Additional factors may contribute to the observed A site occupancy, such as the entropic gain afforded by a disordered structure at high temperatures. Charge differences are unlikely to be responsible for the observed structure, as our calculations using a charge-based tolerance factor predict the B site ordered *x* = 1 structure to be the most stable.^48^

### Magnetic Characterization

**Figure 2:**
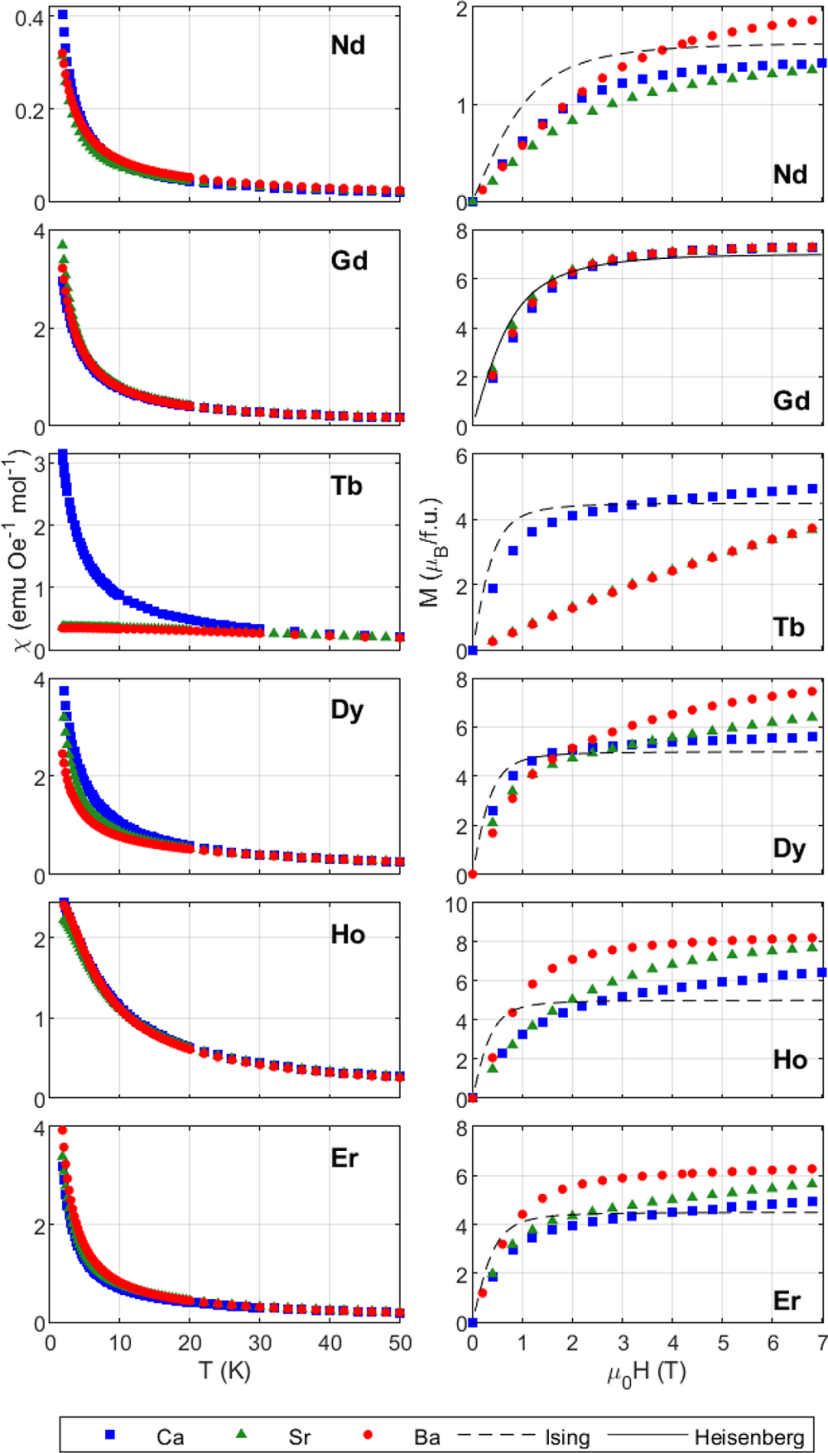
(Left) ZFC magnetic susceptibility χ in a field of 0.1 T for A_2_LnSbO_6_, where A = {Ba, Sr, Ca} and Ln = {Nd–Er}. Shown here are the measured responses for the ordered (*x* = 1.0) samples of Ca_2_HoSbO_6_ and Ca_2_ErSbO_6_. None of the χ-curves exhibit ordering features or anomalies except for Ba_2_TbSbO_6_, which has a shallow peak at 3.50 K (see Figure S9). The FC magnetic susceptibility was also measured and is not shown since no hysteresis was observed for any of the compounds except Ba_2_TbSbO_6_, which is examined in the Supplementary Info. (Right) Isothermal magnetization *M*(*H*) at 2 K versus the applied magnetic field μ_0_*H* for A_2_LnSbO_6_ (A = {Ba, Sr, Ca} and Ln = {Nd–Er}). Predictions for paramagnetic Heisenberg and Ising spins are shown as black solid and dashed lines, respectively. All Gd compounds are well approximated by Heisenberg spins. The site-disordered Ca_2_DySbO_6_ and Ca_2_TbSbO_6_, and ordered Ca_2_ErSbO_6_, saturate close to the value for Ising spins, 1/2*g*_*J*_*J*.

Magnetic susceptibility χ measurements were conducted in an applied field of 0.1 T in both zero-field cooled (ZFC), Figure 2, and field-cooled (FC) conditions. None of the compounds exhibit features indicative of a long-range ordering transition down to 2 K. Ba_2_TbSbO_6_ contains a shallow peak in χ at 3.5 K, which could be attributable to the onset of short-range fluctuations. There is no irreversibility observed in the ZFC/FC measurements with the exception of Ba_2_TbSbO_6_ below 10 K, Figure S9, for which a 0.1% deviation is observed.

Curie–Weiss fits, Table 2, of the inverse susceptibility χ^–1^ were carried out for each compound at low temperatures to avoid the contribution of low-lying excited states.^14^ Due to the observation of magnetic ordering in Ba_2_TbSbO_6_ at *T* = 3.5 K, fitting to the Curie–Weiss law was carried out at higher temperatures for the Tb^3+^-containing materials. Since the magnetic susceptibility was measured in the field region where *M* scales linearly with *H*, the Heisenberg approximation of free-spins is valid and μ_eff_ should be close to the theoretical prediction.^50^ The fit moments, Table 2, are in broad agreement with the theoretical moments, consistent with previous reports.^16,28,29^ The fit μ_eff_ for all Gd compounds is slightly greater than the 7.94 μ_*B*_/f.u. prediction, as has been observed in the similar series Li_3_Ln_3_Te_2_O_12_.^14^

**Table 2:** Fit Curie Temperature Θ and Effective Magnetic Moment μ_eff_ of A_2_ LnSbO_6_ (A = {Ba, Sr, Ca} and Ln = {Nd–Er})^a^

Ln	A	Θ (K)	*f>*	μ_eff_ (μ_B_/f.u.)	μ_theory_ (μ_B_/f.u.)	*T* fit (K)	*R*^2^	*J*_ex_ (K)	*D*_*nn*_ (K)	*D*_*nn*_/*J*_ex_
Nd	Ba	–6.8(3)	3.8	3.33(1)	3.62	20–50	0.9999	1.7(1)	0.0313(1)	0.2
	Sr	–9.0(7)	5.0	3.16(3)		20–50	0.9992	2.2(3)	0.0301(1)	0.1
	Ca (*x* = 0)	–6.1(7)	3.4	3.03(3)		20–50	0.9993			
Gd	Ba	–0.777(3)	1.9	8.18(1)	7.94	8–50	1.00000	0.146(1)	0.194(1)	1.3
	Sr	–0.51(1)	1.3	8.16(1)		8–50	1.00000	0.076(4)	0.205(1)	2.7
	Ca (*x* = 0)	–0.923(8)	=1.8	8.18(1)		8–50	1.00000			
**Tb**	Ba	–12.1(5)	=3.5	9.86(1)	9.72	30–300	0.9999	3.0(2)	0.284(1)	0.1
	Sr	–11.4(4)	6.3	9.82(1)		30–300	0.9999	2.8(2)	0.300(1)	0.1
	Ca (*x* = 0.215(3))	–12.7(9)	7.1	10.23(1)		30–300	0.9998			
Dy	Ba	–10.3(2)	5.7	11.14(4)	10.65	8–50	0.9995	2.48(8)	0.365(3)	0.2
	Sr	–8.5(1)	4.7	10.90(1)		15–50	1.00000	2.03(3)	0.372(1)	0.2
	Ca (*x* = 0.552(3))	–8.5(2)	4.7	10.22(3)		8–50	0.9997			
Ho	Ba	–2.10(5)	1.2	10.37(1)	10.61	5–50	0.9999	0.45(2)	0.318(1)	0.7
	Sr	–2.78(7)	1.5	10.66(1)		5–50	0.9999	0.61(3)	0.358(1)	0.6
	Ca (*x* = 1.0)	–2.51(6)	1.4	10.64(2)		5–50	0.9998	0.53(2)	0.381(1)	0.7
	Ca (*x* = 0.847(2))	–3.20(9)	1.8	10.45(1)		5–50	1.00000	0.71(3)	0.368(1)	0.5
Er	Ba	–6.0(3)	3.3	9.53(4)	9.58	20–50	0.9995	1.4(2)	0.270(2)	0.2
	Sr	–6.0(3)	3.3	9.63(4)		15–50	0.9996	1.4(1)	0.294(2)	0.2
	Ca (*x* = 1.0)	–8.8(3)	4.9	9.69(4)		20–50	0.9997	2.1(2)	0.318(3)	0.2

a The inverse magnetic susceptibility χ^–1^ was fit using linear regression to the Curie–Weiss law over the specified temperature range. Estimates for the nearest-neighbor exchange *J*_ex_ and the dipolar interaction *D*_*nn*_ are computed as described in the SI using the method from reference (49). The Ln = Gd compound data are taken from our previous report, where heat capacity indicated ordering of Ca_2_GdSbO_6_ at 0.52 K and a lack of ordering to 0.4 K for Ba_2_GdSbO_6_ and Sr_2_GdSbO_6_.^26^ The site disorder for each Ca compound is listed according to the formula [Ca_(1+*x*)_Ln_(1–*x*)_]_A_[Ca_(1–*x*)_Ln_*x*_]_B_SbO_6_, with *x* = 1.0 corresponding to an ordered *fcc* structure of Ln^3+^ on the B site and *x* = 0 corresponding to a disordered structure of Ln^3+^ shared equally with Ca^2+^ on the A site.

All compounds exhibit negative Curie–Weiss temperatures, Table 2, indicative of antiferromagnetic superexchange. For all Ln, the lower limit for the frustration index *f* = |Θ_*CW*_|/*T*_0_ exceeds 1, where *T*_0_ is the magnetic ordering temperature, and when no magnetic ordering is observed *T*_0_ is assumed to be the lowest temperature measured, i.e., *T*_0_ = 2 K, or *T*_0_ = 0.5 K in the case of Gd-containing samples. Materials with *f* > 10 are considered “strongly” frustrated^51^ and so lower temperature measurements are therefore required to gain a more accurate indication of the level of frustration. Heat capacity measurements down to 0.3 K showed a lack of ordering in Ba_2_GdSbO_6_ and Sr_2_GdSbO_6_, while the disordered Ca_2_GdSbO_6_ orders at 0.52 K.^26^ Mean-field estimates for the *nn* exchange *J*_ex_ and dipolar interaction *D*_*nn*_, Table 2, indicate that *J*_ex_ exceeds *D*_*nn*_ in all the materials except Ln = Gd.

Figure 2 depicts the isothermal magnetization *M*(*H*) of A_2_LnSbO_6_ (A = {Ba, Sr, Ca} and Ln = {Nd–Er}) at 2 K. All Ln = Gd compounds, including the site-disordered [CaGd]_A_[Ca]_B_SbO_6_, agree with the predicted magnetization for free Heisenberg spins, although they exhibit a slightly lower magnetization at low fields (μ_0_*H* = 0–2 T) due to *nn* antiferromagnetic fluctuations.^26^ The Ln compounds with unquenched orbital angular momentum exhibit single-ion anisotropy, as they do not saturate at either the maximum Ising (0.5*g*_*J*_*J*) or Heisenberg predictions (*g*_*J*_*J*).

Changes to the chemical pressure via tuning of the A site cation appear to have a small effect on χ, Figure 2, with the exception of Ln = {Tb,Dy}. All other Ln compounds have similar magnetic susceptibilities for temperatures of 2 K and above. Both Ca_2_TbSbO_6_ and Ca_2_DySbO_6_ exhibit site disorder and demonstrate increased magnetic susceptibility at low temperatures relative to the *fcc* A = {Ba, Sr} versions, indicating that disorder may promote ferromagnetic interactions rather than chemical pressure.

The isothermal magnetization varies with the A site cation for each Ln with single ion anisotropy, likely due to change in the oxygen coordination and crystal electric field effect. The local site symmetry for the rare earths changes with the alkaline earth cation and could play a role in the magnetism of the Kramers versus non-Kramers ions. Note that for Ln = Tb and Ln = Dy, where significant differences in the magnetic susceptibility χ are observed, there are also significant differences in the isothermal magnetization for A = {Ba, Sr} versus A = Ca.

### Deviations from Curie–Weiss Behavior

**Figure 3:**
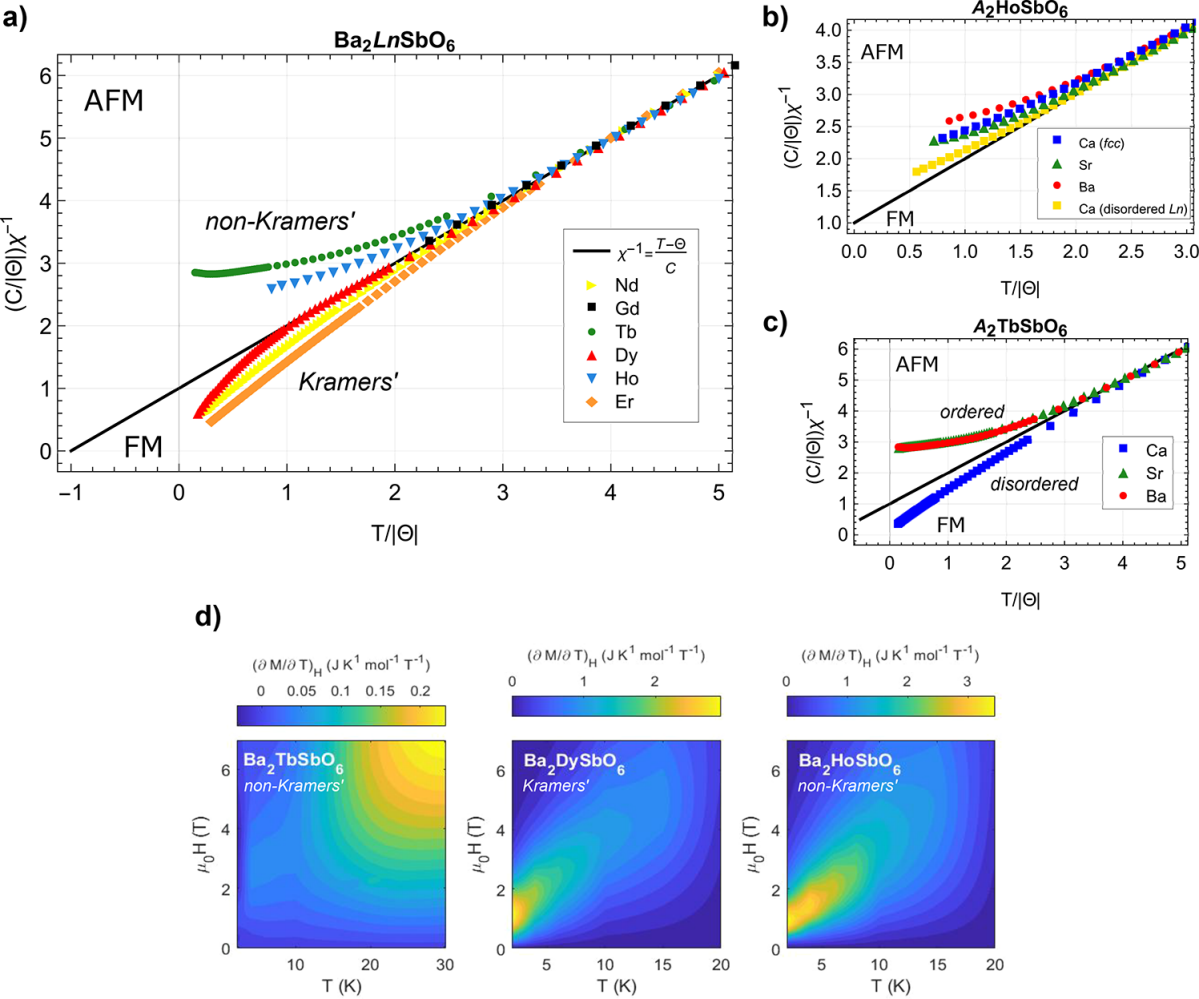
Effects of tuning deviations in the magnetic susceptibility via the magnetic Ln ion, chemical pressure, and disorder via the A site cation. (a) Dimensionless inverse magnetic susceptibility χ^–1^ of Ba_2_LnSbO_6_. Both axes are scaled to be dimensionless using the appropriate factors of the Curie constant *C* and Curie temperature Θ from the Curie–Weiss fit for each material. The predicted dimensionless susceptibility for paramagnetic (uncoupled) spins is shown by the black line, (*C*/|Θ|)χ^–1^ = *T*/|Θ| + 1. Positive (negative) deviations from this line indicate values of the magnetic susceptibility that are smaller (greater) than that expected for uncoupled spins, which can be an indication of antiferromagnetic (ferromagnetic) short-range fluctuations above the ordering temperature. (b) Inverse magnetic susceptibility χ^–1^ of A_2_HoSbO_6_, where A = {Ba, Sr, Ca}. All ordered compounds have the Ho ion ordered on an *fcc* magnetic sublattice (B sites). The disordered Ca_2_HoSbO_6_ has 84.7(2)% of Ho ions distributed on the B site and the remaining 15.3(2)% randomly disordered with Ca^2+^ on the A sites. (c) Inverse magnetic susceptibility χ^–1^ of A_2_TbSbO_6_, where A = {Ba, Sr, Ca}. The disordered Ca-based compound exhibits FM deviations compared to the AFM deviations for A = {Ba, Sr}. (d) Temperature derivative of the magnetization (∂*M*/∂*T*)_*H*_ = (∂*S*/∂*H*)_*T*_ for Ba_2_LnSbO_6_ for two non-Kramers ions, Ln = {Tb^3+^, Ho^3+^}, and one Kramers ion, Ln = Dy3+. Representative of other Kramers ions in the *fcc* A_2_LnSbO_6_ (A = {Ba, Sr}) family, the Dy compound exhibits a peak in (∂*M*/∂*T*)_*H*_ at the lowest temperatures and lower fields. In contrast, the non-Kramers Tb and Ho compounds have a maximum in (∂*M*/∂*T*)_*H*_ at finite temperatures and higher fields, indicating possible CEF effects and/or the importance of antiferromagnetic exchange between spins. Note the difference in color bar scales between the compounds.

To enable a comparison across compounds, deviations from the Curie–Weiss law were investigated using a dimensionless plot of χ^–1^, Figure 3a–c). As in refs (52) and (53), we write the Curie–Weiss law in the dimensionless form as follows:
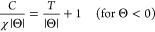
2where Θ is the Curie temperature and *C* is the Curie constant. In these dimensionless units, paramagnetic behavior corresponds to the linear relationship of  with  with an *y*-intercept of 1. Positive or negative deviations from linearity can indicate antiferromagnetic or ferromagnetic short-range fluctuations, respectively. Deviations from Curie–Weiss behavior are also possible for single-ion systems that exhibit the gradual population of magnetic excited states with increasing temperature.

#### Effect of Tuning the Ln^3+^ ion: Kramers v. Non-Kramers Ions

The dimensionless χ^–1^ plot, Figure 3a (and Figure S10), shows that the Gd-based compounds remain paramagnetic down to 1.8 K, indicating minimal deviations from Curie–Weiss behavior. For the Ln^3+^ ions with unquenched orbital angular momentum, however, a pattern emerges in which the non-Kramers ions tend toward antiferromagnetic deviations, while the Kramers ions tend toward ferromagnetic deviations.

This has implications for the magnetocaloric performance of these materials. For example, Figure 3d depicts the temperature derivative of the magnetization (∂*M*/∂*T*)_*H*_ = (∂*S*/∂*H*)_*T*_ from which the magnetic entropy change Δ*S*_m_ is derived. Dy, a Kramers ion, exhibits a maximum in (∂*M*/∂*T*)_*H*_ at the lowest temperatures measured and a small (0.5–1 T) field, in agreement with the ferromagnetic short-range fluctuations in χ^–1^. Instead, for non-Kramers ions (e.g., Tb, Ho), (∂*M*/∂*T*)_*H*_ is maximized at finite temperatures and fields, Figure 3d.

Two possible explanations for the positive deviations in χ^–1^ of non-Kramers ions include (1) antiferromagnetic short-range fluctuations between spins and/or (2) crystal electric field (CEF) effects. In the first case, polarization of spins at any temperature would require a larger external field to counteract the energetically preferable anti-alignment between *nn* spins. *M*(*H*) measurements, Figure 2, support this explanation, as *M*(*H*) for Ln = {Tb, Ho} underestimates the prediction for Heisenberg and Ising spins at low fields (μ_0_*H* = 1–3 T).

A second possible cause is the CEF effect. In ref (28), positive deviations of χ^–1^ of Ba_2_HoSbO_6_ from Curie–Weiss behavior at low temperature are attributed to a gradual depopulation of excited states and strong quantum fluctuations.^28^ In this case, one might expect a maximum in (∂*M*/∂*T*)_*H*_ at finite temperatures and fields, as (1) a finite temperature is required to access these excited, magnetic states and (2) an applied magnetic field can alter the energy difference between these states. This behavior is observed for all Ho- and Tb-containing compounds in this work, e.g., those in Figure 3d.

#### Effect of Chemical Pressure: A = {Ba, Sr}

The effect of chemical pressure on the deviations from Curie–Weiss behavior in the ordered *fcc A*_2_LnSbO_6_ compounds, Figure 3a–c) (and Figure S10), depends on the Ln ion. While the dipolar interaction, crystal electric field parameters, and superexchange interactions need to be characterized to make definitive claims about the changes with chemical pressure, some trends between the different Ln ions can be observed.

First, for the Kramers ions Ln = {Nd, Dy}, the cubic A = Ba^2+^ version exhibits decreased ferromagnetic deviations compared to those with A = Sr^2+^. For Ln = Er, a different behavior is observed in which the ferromagnetic deviations from the Curie–Weiss law increase with the increasing radius of the A cation. One possible explanation for the change for A = Ba^2+^ relative to the A = {Sr^2+^, Ca^2+^} counterparts is the increased orbital overlap of 4f electrons due to the cubic lattice, resulting in enhanced superexchange. Another could be the alteration of the ground state and low-lying excited states via the CEF effect. All three types of *fcc* compounds Ln = {Nd, Dy, Er} have an estimated ratio of the dipolar interaction and superexchange *D*_*nn*_/*J*_ex_ on the order of 0.2, which could contribute to their similarities in short-range fluctuations, Table 2.

Second, for the non-Kramers Ln = Ho compounds, the cubic A = Ba^2+^ compound also exhibits the largest deviations, followed by Ca and then Sr (Figure 3c). This could be due to the interplay of orbital overlap and the *nn* distance in determining the magnitude of the superexchange interaction as well. For Ln = Tb, there is no observable difference in the magnitude and temperature onset of the positive deviations in the inverse magnetic susceptibility for A = {Ba, Sr}. The dipolar to superexchange ratios in these systems are more distinct for Ln = {Ho, Tb}, where *D*_*nn*_/*J*_ex_ ∼ 0.6 and ∼0.1, respectively, which could suggest that different interactions might be more relevant in explaining the effect of chemical pressure on the magnetic susceptibility deviations.

#### Effect of Site Disorder: A = Ca

Figure 3c depicts the dimensionless χ^–1^ of A_2_HoSbO_6_ (A = {Ba, Sr, Ca}) for a site-disordered version of A = Ca in which 84.7(2)% of the Ho ions lie on the B site and an ordered version in which the Ho ions are distributed on an *fcc* lattice. The reduced antiferromagnetic deviations of the disordered compound relative to the *fcc* version support the hypothesis that disorder promotes ferromagnetic short-range fluctuations. This effect is more pronounced for Ln = Tb, Figure 3b), as both the *fcc* Sr and Ba versions exhibit positive deviations from Curie–Weiss behavior while the site-disordered Ca version exhibits net negative deviations.

### Magnetocaloric Effect

**Figure 4:**
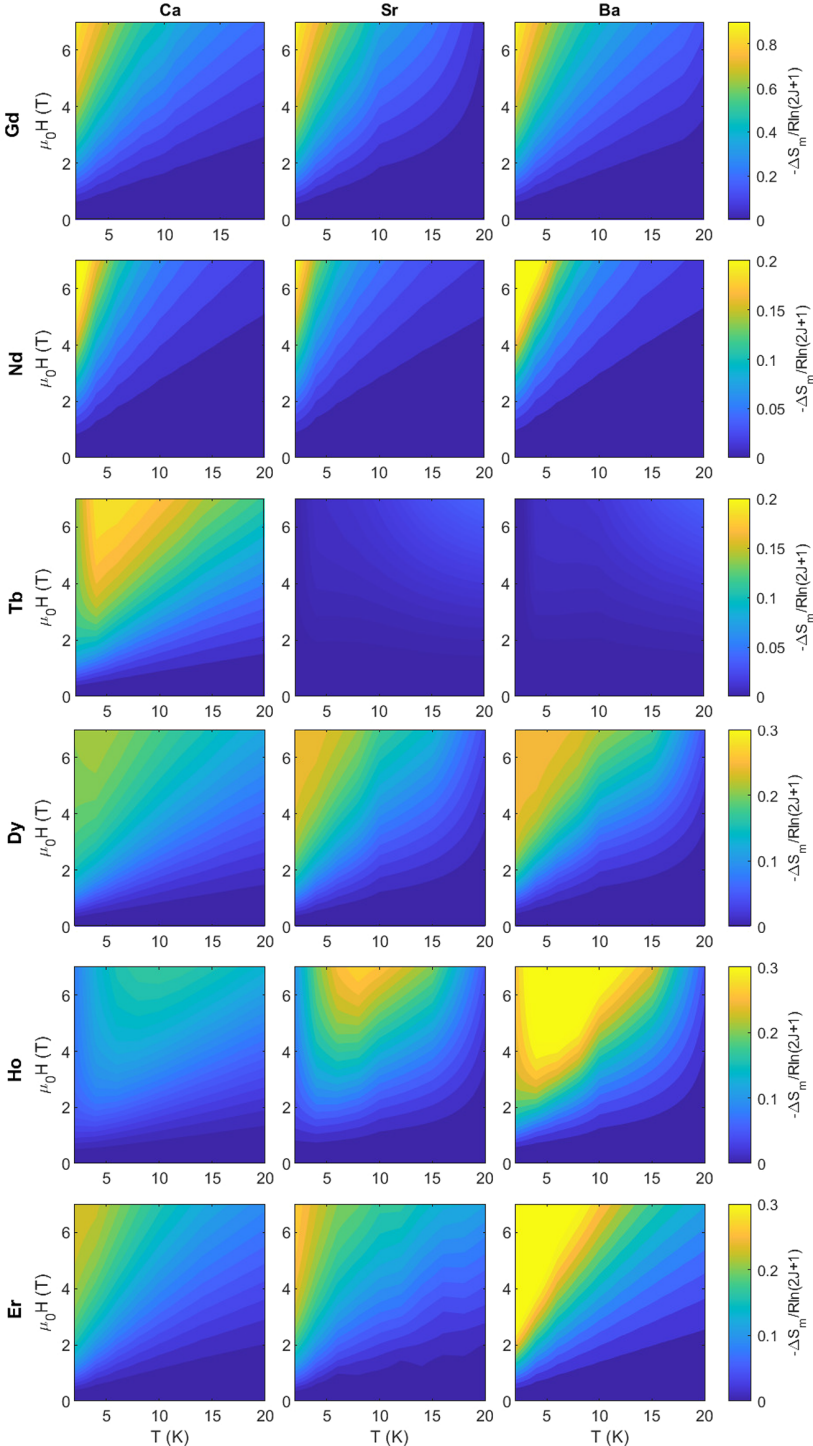
Isothermal magnetic entropy change Δ*S*_*m*_ for A_2_LnSbO_6_, where A = {Ba, Sr, Ca} and Ln = {Gd, Nd, Tb, Dy, Ho, Er}, as a function of temperature *T* and applied magnetic field μ_0_*H*. All color maps are scaled by the maximum entropy change for paramagnetic Heisenberg spins with total angular momentum *J*, *R*ln(2*J* + 1). Note the difference in the color bar scales, as Ln = Gd attains a maximum magnetic entropy change of 0.9*R*ln(2*J* + 1) while Nd and Tb attain up to 0.2*R*ln(2*J* + 1). The site disorder *x* of the Ca_2_LnSbO_6_ compounds measured here are listed in Table 3.

The magnetocaloric effect of each material was studied under applied fields of 0.2–7.0 T and temperatures of 2–20 K. The isothermal magnetic entropy change Δ*S*_m_ was measured from the isothermal magnetization, Figure 4. Key values are summarized in Table 3.

**Table 3:** Magnetic Entropy Change Δ*S*_m_ of A_2_LnSbO_6_ (A = {Ba, Sr, Ca}, Ln = Nd–Er) at 2 K for Applied Fields of 7 and 2 T^a^

Ln	*x* (A = Ca)	Ba_2_LnSbO_6_	Sr_2_LnSbO_6_	Ca_2_LnSbO_6_
–Δ*S*_m_ at 7 T (J/K·mol)				
Nd	0	5.46	4.07	4.65
Gd	0	15.84	15.83	15.35
Tb	0.215(3)	–0.08	0.04	2.96
Dy	0.552(3)	5.83	5.79	4.86
Ho	0.847(2)	6.22	2.46	1.92
Er	1.0	10.08	6.30	5.50
–Δ*S*_m_ at 2 T (J/K·mol)				
Nd	0	1.12	0.95	1.22
Gd	0	6.61	7.04	6.07
Tb	0.215(3)	0.002	0.02	2.34
Dy	0.552(3)	4.23	4.09	3.57
Ho	0.847(2)	4.47	1.08	1.34
Er	1.0	6.03	4.06	3.63

a For reference, Gd_3_Ga_5_O_12_ exhibits Δ*S*_m_ values of −13.0 and −4 J/K/mol at 7 and 2 T, respectively.^14^ The site disorder for each Ca compound is listed according to the formula [Ca_(1+*x*)_Ln_(1–*x*)_]_A_[Ca_(1–*x*)_Ln_*x*_]_B_SbO_6_, with *x* = 1.0 corresponding to an ordered *fcc* structure of Ln^3+^ on the B site and *x* = 0 corresponding to a disordered structure of Ln^3+^ shared equally with Ca^2+^ on the A site.

**Figure 5:**
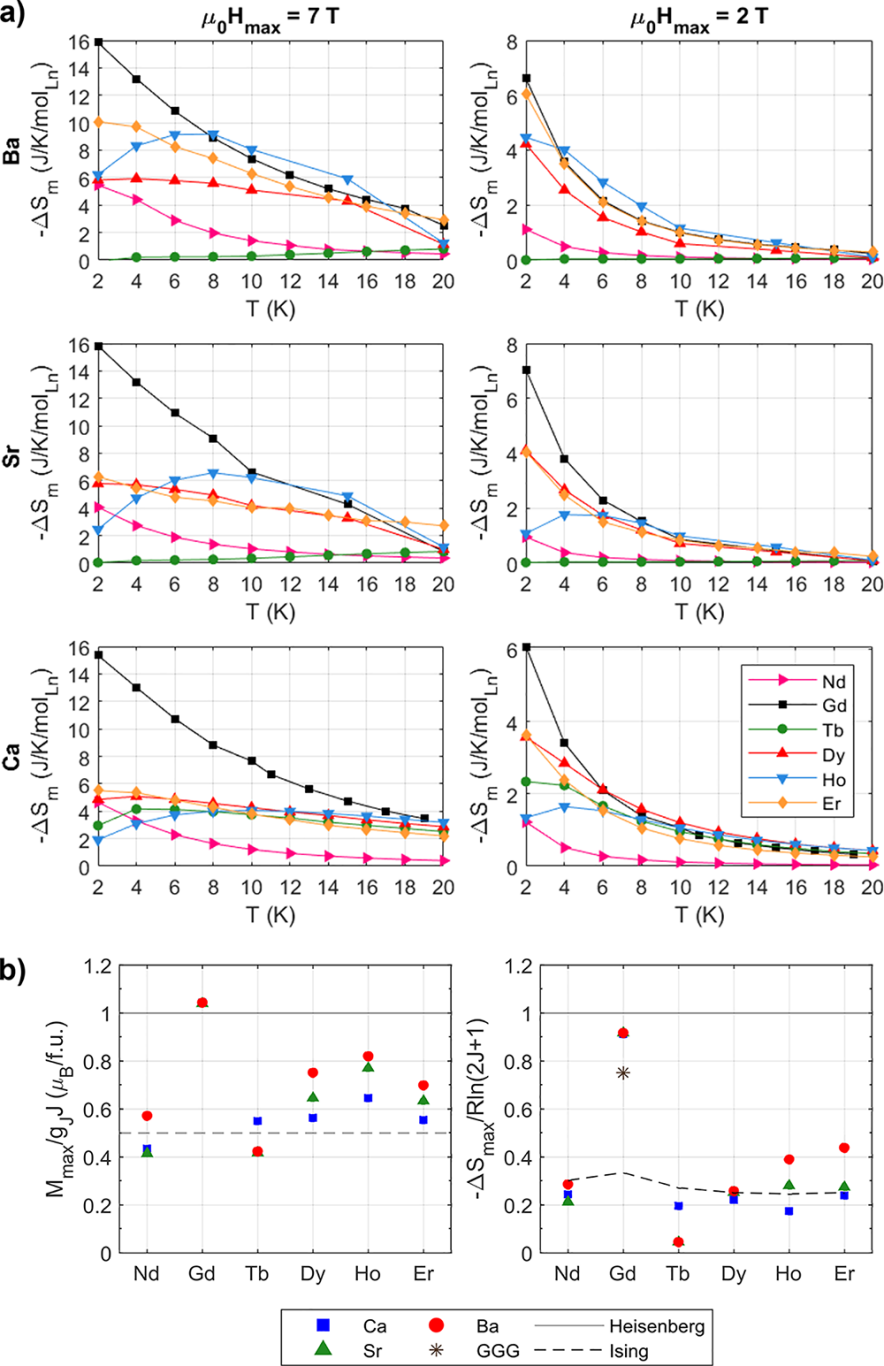
(a) Isothermal magnetic entropy change Δ*S*_*m*_ from starting fields of μ_0_*H* = 7 T (left column) and μ_0_*H* = 2 T (right column) versus temperature *T* for A_2_LnSbO_6_, where A = {Ba, Sr, Ca} and Ln = {Nd–Er}. Note that the Ln = Nd series is shown as pink triangles (compared to yellow in Figure 3a). Solid lines are shown to aid the eye. (b) Maximum measured magnetization *M*_max_ of A_2_LnSbO_6_ (A = {Ba, Sr, Ca} and Ln = {Nd–Er}) achieved at 2 K and 7 T, and maximum measured magnetic entropy change Δ*S*_max_ reached in an applied field of up to 7 T and temperature range 2 to 22 K, scaled as a function of the theoretical values *g*_*J*_*J* and *R*ln(2*J* + 1) for free Heisenberg spins. The max experimental entropy change for Gd_3_Ga_5_O_12_ (GGG) at 7 T and 2 K is provided as a benchmark. *M*_max_ and Δ*S*_max_ for free Heisenberg spins are shown as solid black lines, while those for Ising spins, *M*_max,Ising_ = 1/2*g*_*J*_*J* and −Δ*S*_max,Ising_ = *R*ln 2, are shown as dashed black lines.

Slices of Δ*S*_m_ for A_2_LnSbO_6_ versus temperature at 7 and 2 T are shown in Figure 5a. The Ln = Gd compounds are the highest performing, reaching up to −16 J/K/mol_Ln_, at 7 T and 2 K compared to −13.0 J/K/mol_Ln_ for Gd_3_Ga_5_O_12_. Ba_2_ErSbO_6_ is also a promising magnetocaloric material, with entropy changes of −10 and −6 J/K/mol_Ln_ at 2 T, on par with the 2 T value for Gd_3_Ga_5_O_12_.^54^ This can likely be explained by the free spin nature of Ba_2_ErSbO_6_ reported by Calder et al.^29^ Unlike the Ga-based garnets Ln_3_Ga_5_O_12_ in which Ln = {Tb, Dy} outperform Gd at low (μ_0_*H* ≤ 2 T) fields,^14^ Gd remains the ideal Ln ion at 2 K across all fields, Figure S11. However, Ba_2_HoSbO_6_ outperforms Ba_2_GdSbO_6_ at *finite* temperatures: 8–16 K at 7 T and 4–10 K at 2 T. The peak in −Δ*S*_m_ with temperature for Ba_2_HoSbO_6_ in Figure 5a is likely due to the population of its low-lying magnetic, excited states, the first of which is 10 K.^28^ The *fcc* Tb compounds show a very small magnetic entropy change starting at 10 to 20 K, continuing up to 30 K. This could be a result of polarizable defects in a short-range correlated or long-range ordered state or the thermal population of excited states.

Figure S11 depicts Δ*S*_m_ as a function of the applied field. All Ln except Nd and Gd reach a plateau at fields less than 7 T, indicating that some compounds, namely Ba_2_LnSbO_6_ (Ln = {Dy, Ho, Er}), could be used as magnetocaloric materials in smaller fields. The site-disordered Ca compounds (Ln = {Gd–Dy}) plateau in Δ*S*_m_ at smaller fields (∼3–4 T), consistent with ferromagnetic short-range fluctuations suggested by Figure 3.

The related *fcc* [La*A*]_*A*_CoNbO_6_ series was found to have the lowest ordering temperature for the largest A site cation, Ba, compared to Sr and Ca due to enhanced frustration afforded by a uniform lattice.^20^ Thus, it is possible that the Ba series presented here could also enabling cooling down to lower temperatures than its Sr and Ca counterparts, which we found to the be the case for Ln = Gd^3+^.^26^

## Conclusion

This work investigates the effect of tuning the magnetic Ln ion and chemical pressure (A site cation) on the structural, magnetic, and magnetocaloric properties of *fcc* lanthanide oxides. All A = {Ba^2+^, Sr^2+^} compounds have an *fcc* magnetic sublattice with antiferromagnetic superexchange. The A = Ca^2+^ series is reported for the first time and exhibits tunable site-disorder with controlled cooling in the solid-state synthesis.

With the exception of the Tb-containing phases, none of the materials exhibit long-range order down to 1.8 K, signaling the possibility of magnetic refrigeration down to this temperature. Bulk magnetic measurements suggest that the magnetocaloric effect in frustrated *fcc* magnets is optimized in the presence of minimal short-range correlations, as evidenced by the nearly free-spin A_2_GdSbO_6_ materials;^26^ for Ln ions with quenched orbital angular momentum (i.e., Gd); and in materials with uniform magnetic sublattices (A = Ba^2+^), possibly due to decreased single-ion anisotropy.

The measurements indicate a possible link between the magnetic entropy change and the apparent short-range fluctuations in the system. This work suggests that the magnetic ground state of the system, determined by the Ln ion and chemical pressure, can be utilized to tune the field-temperature phase space of the magnetocaloric effect through changes to the superexchange, dipolar interactions, and the crystal electric field. The role of disorder in the magnetocaloric effect in this temperature range is still outstanding. For Ln = Tb^3+^, the presence of site disorder in Ca_2_TbSbO_6_ enables a magnetic entropy change 4.5× greater than Sr_2_TbSbO_6_ and Ba_2_TbSbO_6_. Low-temperature μSR experiments, heat capacity in field, and magnetic neutron scattering will be key in elucidating the role of disorder in the magnetic ground state and magnetocaloric properties of these rare-earth *fcc* systems.

## Supporting Information Available

Refined structural parameters, analysis of magnetic susceptibility, magnetocaloric effect calculations, and further magnetic characterization (PDF)
Data associated with this publication can be found at: https://doi.org/10.17863/CAM.96860.
