# Supplementary material for: Magnetic and Magnetocaloric Properties of the A2LnSbO6 Lanthanide Oxides on the Frustrated fcc Lattice
Source: Inorg Chem. 2023 Jun 16;62(26):10317–28. doi: 10.1021/acs.inorgchem.3c01137 (PMC10324300; doi:10.1021/acs.inorgchem.3c01137)
Supplement: Supplementary file 1 — ic3c01137_si_001.pdf [file ic3c01137_si_001.pdf]

Supplementary Information: Magnetic and magnetocaloric properties of the A_2LnSbO$_6$ lanthanide oxides on the frustrated *fcc* lattice

EliseAnne C. Koskelo,[†,§] Nicola D. Kelly,[†,‖] Liam A. V. Nagle-Cocco,[†] Joshua D. Bocarsly,[†,‡] Paromita Mukherjee,[†] Cheng Liu,[†] Qiang Zhang,[¶] and Siân E. Dutton[*,†]

† *Department of Physics, University of Cambridge, Cambridge, CB3 0HE, United Kingdom*

‡ *Yusuf Hamied Department of Chemistry, University of Cambridge, Cambridge, CB2 1EW, United Kingdom.*

¶ *Neutron Scattering Division, Oak Ridge National Laboratory, Oak Ridge, Tennessee, USA*

§ *Current address: Department of Physics, Harvard University, Cambridge, MA 02138, USA.*

‖ *Current address: : Department of Chemistry, University of Oxford, Inorganic Chemistry Laboratory, South Parks Road, Oxford OX1 3QR, United Kingdom*

E-mail: sed33@cam.ac.uk

Refined Structural Parameters

This section contains the refined structural parameters for the Ca_2LnSbO_6 series at 100 K, and room temperature refinements of Ba_2LnSbO_6 and Sr_2LnSbO_6 as described in the text.

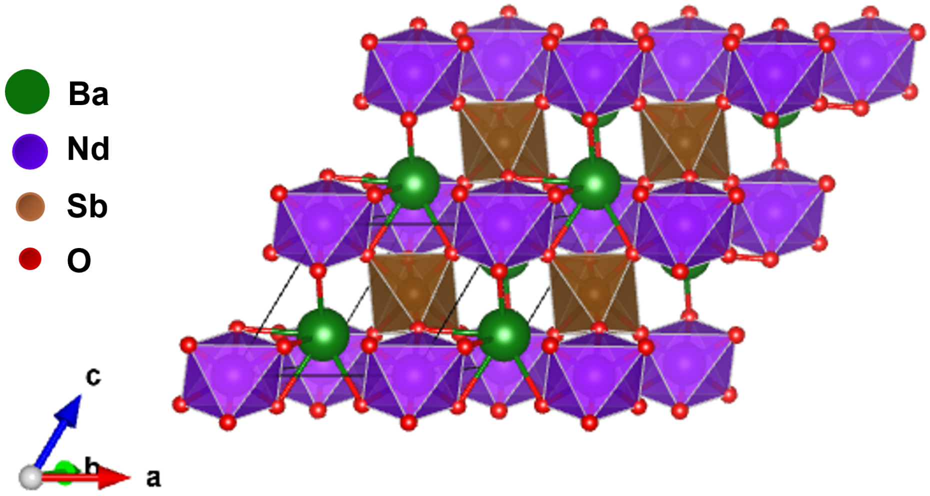

Figure S1: Refined crystal structure of Ba_2NdSbO_6 (space group $R\bar{3}$).

Table S1: Structural parameters of Ca_2LnSbO_6 where Ln ={Nd^{3+}, Gd^{3+}, Tb^{3+}, Dy^{3+}, Ho^{3+}, Er^{3+}} at 100 K determined from high resolution x-ray synchrotron measurements at the I11 beamline. Refinements were carried out in the space group $P2_1/n$, with Ca_1/Ln_2 on the 4e sites (x, y, z), Ln_1/Ca_2 on the 2d sites $(0.5, 0, 0)$, Sb on the 2c sites $(0, 0.5, 0)$, and O(1),O(2), and O(3), on the 4e sites (x, y, z). In the case where a small impurity peak was present near $2\theta = 29°$, an impurity phase of Ln_3SbO_7 was fit using the reported structure.[1–3] Ca_2GdSbO_6 is reported in our previous study, listed here for comparison.[4]

Ln		Nd	Gd	Tb	Dy	Ho	Er
**Ca_2LnSbO_6**							
a (Å)		5.61717(2)	5.58025(2)	5.5656(1)	5.57508(2)	5.58635(1)	5.58117(2)
b (Å)		5.86498(2)	5.84820(2)	5.8353(1)	5.81579(2)	5.79746(1)	5.78314(2)
c (Å)		8.10320(2)	8.07706(2)	8.0587(2)	8.05479(2)	8.03918(1)	8.02415(3)
β (°)		89.7969(2)	90.3253(2)	90.2759(3)	90.0897(2)	90.0869(1)	90.0955(3)
4e: Ca_1/Ln_2	x	0.0161(2)	-0.0174(1)	-0.0134(3)	0.0179(2)	0.0156(4)	0.0179(4)
	y	0.0575(1)	0.05939(7)	0.0598(1)	0.0590(1)	0.0554(1)	0.0546(2)
	z	0.2527(1)	0.25403(8)	0.2506(2)	0.2488(2)	0.2516(4)	0.2512(4)
	Occupancy Ca	0.502(1)	0.5	0.603(1)	0.7821(5)	0.965(1)	0.968(1)
	Occupancy *Ln*	0.497(1)	0.5	0.397(1)	0.2179(5)	0.035(1)	0.032(1)
	B_{iso} (Å^2)	1.30(3)	0.90(1)	0.59	0.81(2)	0.36(2)	0.44(3)
2d: Ln_1/Ca_2	Occupancy *Ln*	0.005(2)	0.0	0.205(2)	0.564(1)	0.930(2)	0.937(1)
	Occupancy Ca	0.995(2)	1.0	0.795(2)	0.436(1)	0.070(2)	0.063(1)
	B_{iso} (Å^2)	1.57(8)	0.68(8)	0.99	0.21(1)	0.362(7)	0.88(4)
2c: Sb	B_{iso} (Å^2)	1.00(3)	0.54(1)	0.40	1.64(2)	0.4	0.16(3)
4e: O(1)	x	0.273(1)	0.1660(8)	0.312(1)	0.315(1)	0.281(1)	0.276(1)
	y	0.324(1)	0.2149(7)	0.334(1)	0.279(1)	0.316(1)	0.313(1)
	z	0.048(1)	-0.0728(6)	0.044(1)	0.069(1)	0.051(1)	0.048(1)
	B_{iso} (Å^2)	1.86(7)	1.25(5)	0.99	1.45(6)	1.00(6)	0.93(7)
4e: O(2)	x	0.322(1)	0.2089(8)	0.307(1)	0.282(1)	0.325(1)	0.319(1)
	y	0.271(1)	0.1769(7)	0.296(1)	0.321(1)	0.274(1)	0.267(1)
	z	0.434(1)	0.5511(6)	0.463(1)	0.450(1)	0.441(1)	0.438(1)
	B_{iso} (Å^2)	1.86(7)	1.25(5)	0.99	1.45(6)	1.00(6)	0.93(7)
4e: O(3)	x	0.887(1)	1.1205(7)	0.884(1)	0.893(1)	0.893(1)	0.897(1)
	y	0.441(1)	0.4390(7)	0.433(1)	0.446(1)	0.451(1)	0.455(1)
	z	0.220(1)	0.2254(5)	0.232(1)	0.282(1)	0.235(1)	0.229(1)
	B_{iso} (Å^2)	1.86(7)	1.25(5)	0.99	1.45(6)	1.00(6)	0.93(7)
Ln_3SbO_7 (wt%)		-	0.48(1)	0.46(3)	-	-	0.43(3)
R_{wp}		2.4	3.7	3.9	2.5	2.2	2.4
χ^2		5.2	5.8	8.6	5.5	4.6	5.0

Table S2: Structural parameters of Ba_2LnSbO_6 where Ln ={Gd^{3+}, Tb^{3+}, Dy^{3+}, Ho^{3+}, Er^{3+}}. PXRD refinements were carried out in the space group $Fm\bar{3}m$, with Ba on the $8c$ sites $(1/4, 1/4, 1/4)$, Ln on the $4a$ sites $(0, 0, 0)$, Sb on the $4b$ sites $(1/2, 1/2, 1/2)$, and O on the $24e$ sites $(x, 0, 0)$. In the case where a small impurity peak was present near $2\theta = 29°$, an impurity phase of Ln_3SbO_7 was fit using the reported structure.[1–3] B_{iso} were set to the literature values.

$\mathbf{Ba_2}\boldsymbol{Ln}\mathbf{SbO_6}$		Gd	Tb	Dy	Ho	Er
a (Å)		8.47517(2)	8.44895(2)	8.43115(2)	8.41451(2)	8.39599(2)
Ba	B_{iso} (Å^2)	0.67	0.67	0.67	0.51	0.5
Ln	B_{iso} (Å^2)	0.48	0.48	0.48	0.10	0.5
Sb	B_{iso} (Å^2)	0.41	0.41	0.41	0.67	0.5
O	x	0.257(2)	0.239(2)	0.258(2)	0.270(1)	0.238(2)
	B_{iso} (Å^2)	0.76	0.76	0.76	0.76	0.79
Ln_3SbO_7 (wt%)		-	1.11(5)	1.46(5)	1.70(6)	2.26(8)
R_{wp}		11.6	13.2	12.0	10.0	10.6
χ^2		1.41	1.47	1.59	1.41	1.67

Table S3: Structural parameters of Ba_2NdSbO_6. PXRD refinements were carried out in the space group $R\bar{3}$, with Ba on the $2c$ sites (x, x, x), Ln on the $1a$ sites $(0, 0, 0)$, Sb on the $1b$ sites $(1/2, 1/2, 1/2)$, and O on the $6f$ sites (x, y, z). B_{iso} were set to the literature values.

$\mathbf{Ba_2NdSbO_6}$		
a (Å)		6.04006(3)
α (°)		60.1007(3)
Ba	x	0.2492(6)
	B_{iso} (Å^2)	0.67
Ln	B_{iso} (Å^2)	0.48
Sb	B_{iso} (Å^2)	0.41
O	x	0.775(7)
	y	0.208(5)
	z	0.293(6)
	B_{iso} (Å^2)	0.76
Ln_3SbO_7 (wt%)		2.3(1)
R_{wp}		10.5
χ^2		1.28

Table S4: Structural parameters of Sr_2LnSbO_6 where Ln ={Nd^{3+}-Er^{3+}}. PXRD refinements were carried out in the space group $P2_1/n$, with Sr on the $4e$ sites (x, y, z), Ln on the $2d$ sites $(1/2, 0, 0)$, Sb on the $2c$ sites $(0, 1/2, 0)$, and each O, O(1),O(2), and O(3), on the $4e$ sites (x, y, z). In the case where a small impurity peak was present near $2\theta = 29°$, an impurity phase of Ln_3SbO_7 was fit using the structure reported in the literature.[1–3] All Debye Waller factors B_{iso} were kept constant with the literature value.

$\mathbf{Sr_2\mathit{Ln}SbO_6}$		Nd	Gd	Tb	Dy	Ho	Er
a (Å)		5.87403(4)	5.84113(5)	5.83087(4)	5.82129(3)	5.81371(6)	5.80464(5)
b (Å)		5.95944(4)	5.89402(5)	5.87129(1)	5.85661(3)	5.84414(6)	5.82783(5)
c (Å)		8.35781(6)	8.29127(7)	8.26894(5)	8.25172(4)	8.23839(8)	8.22079(7)
β (°)		90.3089(5)	90.2373(7)	90.2179(6)	90.2021(4)	90.1879(7)	90.1628(5)
Sr	x	0.0070(7)	0.0105(5)	0.003(1)	0.003(1)	0.0046(9)	-0.0056(9)
	y	0.0378(2)	0.0346(2)	0.0306(2)	0.0286(2)	0.0283(2)	0.0284(3)
	z	0.2480(6)	0.2489(7)	0.2496(8)	0.2486(7)	0.2479(7)	0.2444(6)
	B_{iso} (Å^2)	0.79	0.79	0.79	0.79	0.83	1.18(3)
Ln	B_{iso} (Å^2)	0.24	0.24	0.24	0.24	0.25	0.24
Sb	B_{iso} (Å^2)	0.39	0.39	0.39	0.39	0.29	0.39
O(1)	x	0.238(3)	0.253(3)	0.273(3)	0.272(3)	0.244(3)	0.282(3)
	y	0.285(3)	0.317(3)	0.315(3)	0.292(3)	0.255(3)	0.293(4)
	z	0.058(2)	0.021(3)	0.030(3)	0.042(3)	0.050(2)	0.0140(3)
	B_{iso} (Å^2)	0.79	0.79	0.79	0.79	0.79	0.79
O(2)	x	0.202(3)	0.190(4)	0.210(3)	0.204(3)	0.198(4)	0.325(3)
	y	0.200(3)	0.761(3)	0.763(3)	0.780(3)	0.194(3)	0.221(3)
	z	0.495(2)	0.042(3)	0.049(2)	0.036(3)	0.501(2)	0.472(3)
	B_{iso} (Å^2)	0.79	0.79	0.79	0.79	0.79	0.79
O(3)	x	-0.087(2)	-0.086(2)	-0.079(2)	-0.086(2)	-0.086(3)	-0.093(3)
	y	0.488(2)	0.486(2)	0.487(2)	0.481(2)	0.500(2)	0.507(2)
	z	0.235(2)	0.239(3)	0.237(3)	0.242(2)	0.233(2)	0.257(3)
	B_{iso} (Å^2)	0.79	0.79	0.79	0.79	0.79	0.79
Ln_3SbO_7 (wt%)		0.60(3)	0.67(4)	1.26(3)	1.06(3)	0.85(3)	1.02(3)
		0.49(3) $Sr_2Sb_{1.4}Sr_{0.6}O_6$					
R_{wp}		10.80	8.68	8.83	8.09	7.53	7.26
χ^2		1.24	1.20	1.16	1.23	1.15	1.49

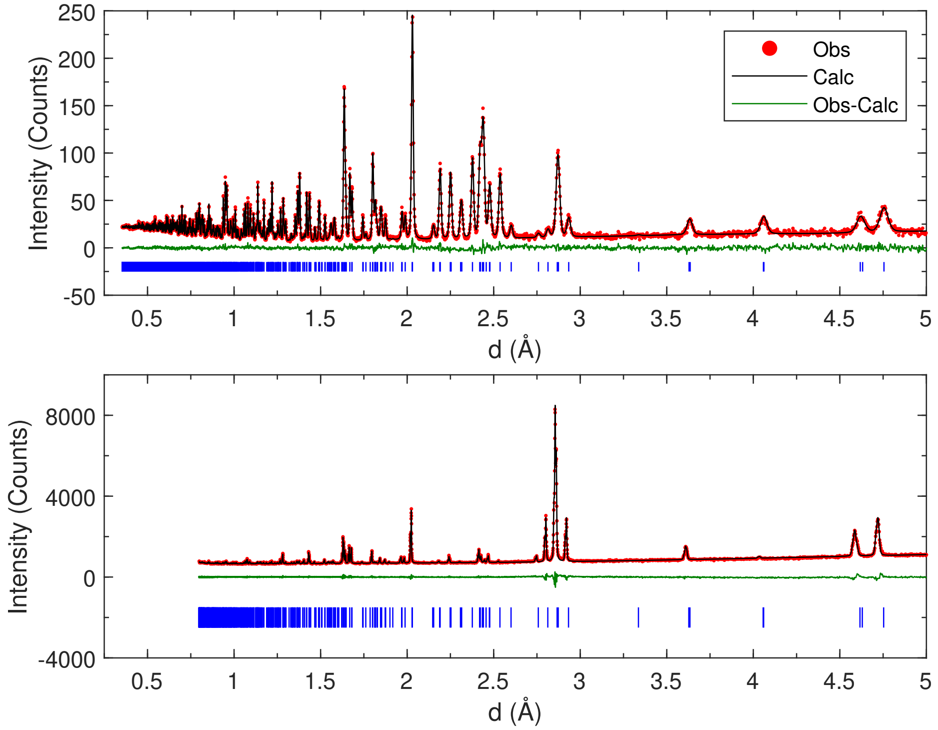

Figure S2: Refined structure of Ca_2NdSbO_6 from a combined Rietveld refinement of room temperature PND and PXRD data. The two peaks at $d \approx 4.63$ and 4.76 Å, corresponding to the reflections (101) and (011), are prominent features of the $Ca_2\mathit{Ln}SbO_6$ compounds with Ln occupancy on the A site.

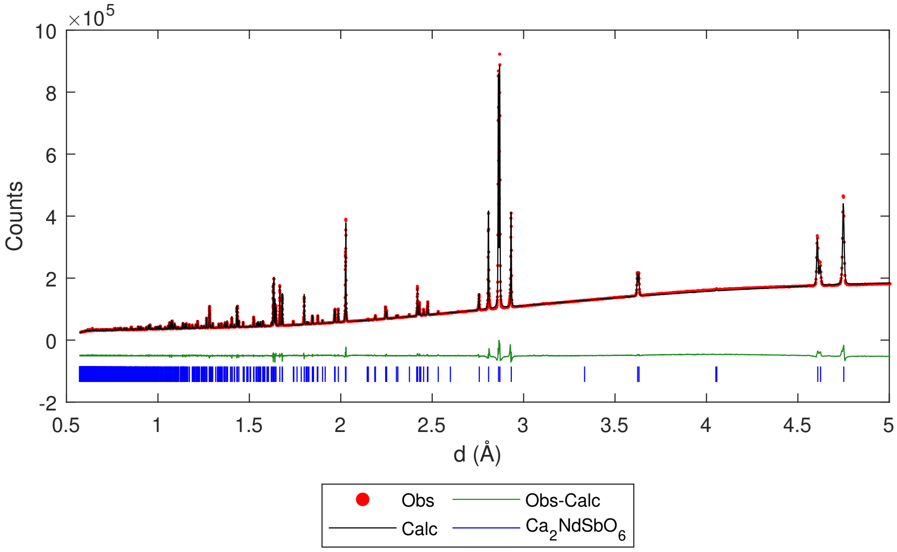

Figure S3: Rietveld refinement of Ca_2NdSbO_6 at 100 K from high-resolution PXRD.

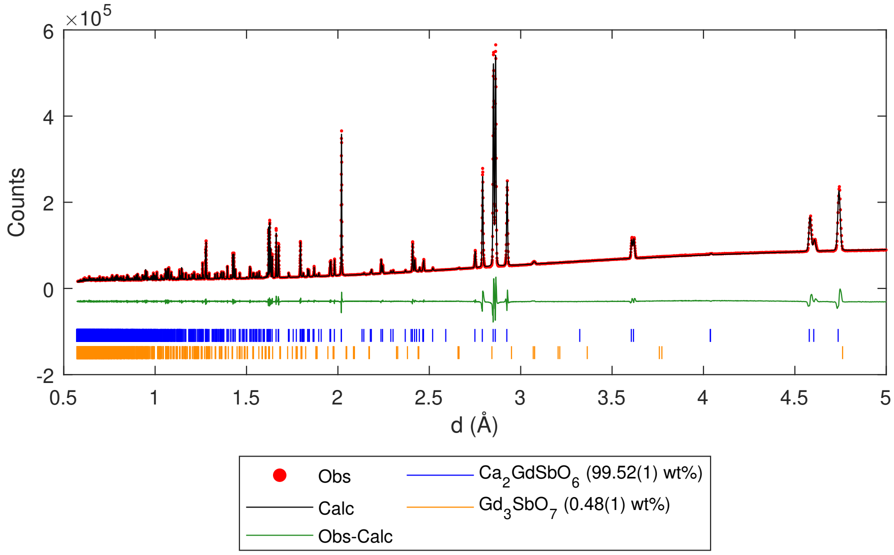

Figure S4: Rietveld refinement of Ca_2GdSbO_6 at 100 K from high-resolution PXRD reported in.[4]

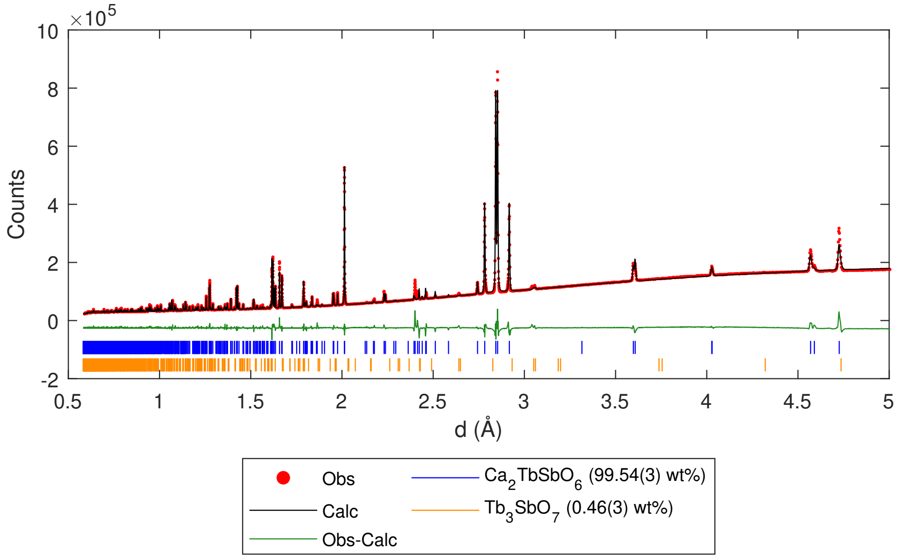

Figure S5: Rietveld refinement of Ca_2TbSbO_6 at 100 K from high-resolution PXRD.

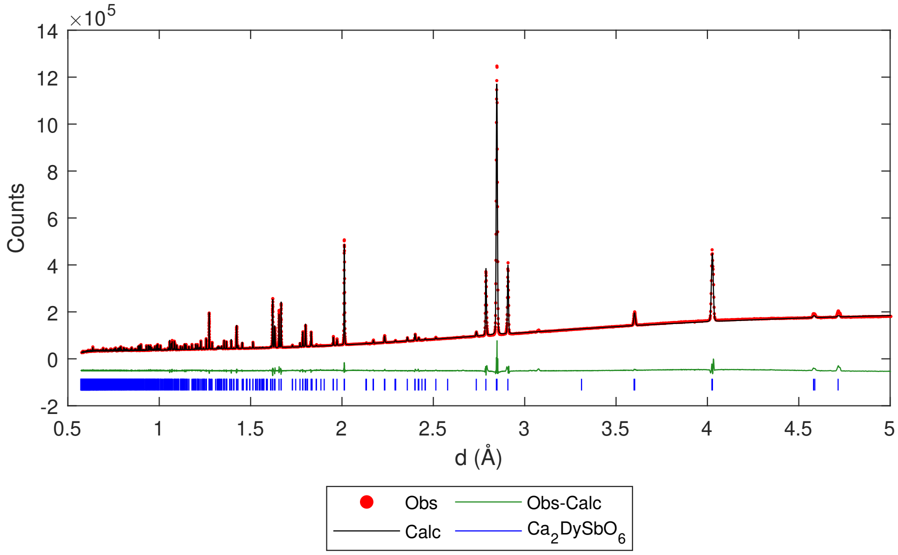

Figure S6: Rietveld refinement of Ca_2DySbO_6 at 100 K from high-resolution PXRD.

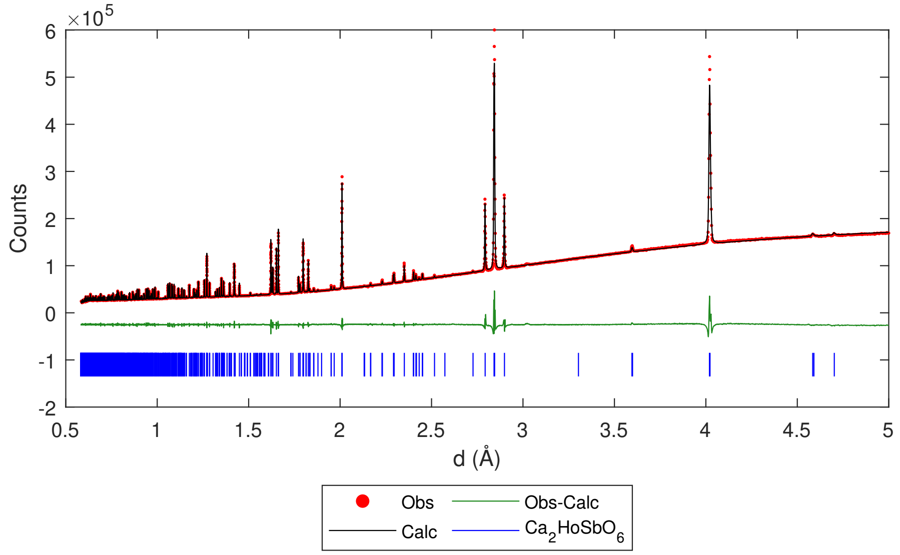

Figure S7: Rietveld refinement of Ca_2HoSbO_6 at 100 K from high-resolution PXRD.

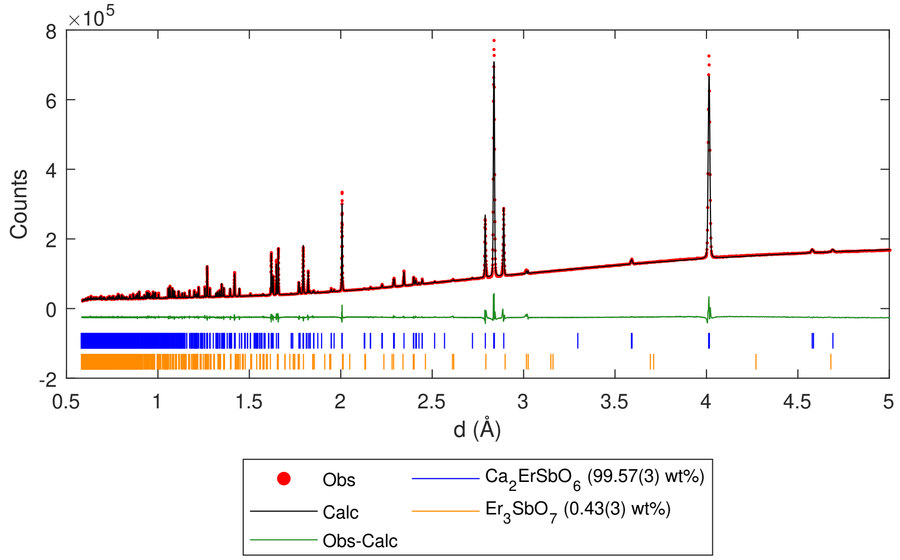

Figure S8: Rietveld refinement of Ca_2ErSbO_6 at 100 K from high-resolution PXRD.

Analysis of Magnetic Susceptibility

The inverse susceptibility $\chi^{-1}(T)$ was fit to the Curie-Weiss law:

$$\chi^{-1} = \frac{T}{C} - \frac{\Theta}{C}, \tag{S1}$$

where Θ is the Curie-Weiss temperature and C is the Curie constant.

For either antiferromagnetic or ferromagnetic exchange, the mean-field approximation enables an estimation of the nn exchange energy J_1 from the Curie-Weiss temperature, Θ, via:[5]

$$J_1 = \frac{-3\Theta}{z(g_J - 1)^2 J(J+1)}, \tag{S2}$$

where z is the number of nearest neighbors for a single magnetic ion with total angular momentum J and g_J is the corresponding Landé g-factor.[6,7]

The effective magnetic moment per spin can be estimated from the Curie constant of a Curie-Weiss fit:[5]

$$\mu_{\text{eff}} = \sqrt{\frac{3k_B C}{N_A}}, \tag{S3}$$

and compared to the theoretical magnetic moment for free spins, $g_J\sqrt{J(J+1)}\mu_B$. Given μ_{eff}, it is possible to obtain an order of magnitude estimate for the dipolar interaction term D_{nn} between spins via:

$$D_{nn} = \frac{\mu_0 \mu_{\text{eff}}^2}{4\pi R_{nn}^3 k_B}, \tag{S4}$$

where μ_0 is the vacuum permeability and R_{nn} is the distance between nearest-neighbor magnetic ions.[7,8] This estimate assumes a mean-field model and Heisenberg spins.

Comparing the nn exchange for different Ln ions can be difficult due to differences in the spin-orbit coupling.[7,9] Here, as in,[9] we compute an order of magnitude estimate for the nn exchange, J_{ex}:

$$J_{\text{ex}} = \frac{-3(\Theta - D_{nn})}{z}, \tag{S5}$$

which accounts for the dipolar interaction contribution to the Curie-Weiss temperature.

Magnetocaloric Effect Calculations

From one of Maxwell's relations, the isothermal field gradient of the magnetic entropy is related to the temperature gradient of the magnetization at constant field via:

$$\left(\frac{\partial S}{\partial H}\right)_T = \left(\frac{\partial M}{\partial T}\right)_H. \tag{S6}$$

The magnetic entropy change for a field $H_{\max}$ relative to zero field, was calculated from the measured $M(H)$ by first computing the temperature derivative of the magnetization using the finite differences approximation:

$$\left(\frac{\partial M(T_i, H)}{\partial T}\right)_H \approx \frac{M(T_{i+1}, H) - M(T_i, H)}{T_{i+1} - T_i}, \tag{S7}$$

and then integrating across fields:

$$\Delta S_m(T_0, H_{\max}) = \int_0^{H_{\max}} \left(\frac{\partial M(T_0, H)}{\partial T}\right)_H dH \tag{S8}$$

The magnetization data $M(H)$ were linearly interpolated along the field direction in steps of 0.1 T prior to extracting the magnetic entropy.

Further Magnetic Characterization

There is a small amount of hysteresis present in Ba_2TbSbO_6 in the ZFC and FC magnetic susceptibilities χ, Figure S9. Both curves exhibit similar curvature, but with a discernible offset below a certain temperature, denoted T_{irr} in Table S5, indicating irreversibility. At small fields ($\mu_0 H < 1$ T), a small peak at 3.5 K is present in both the ZFC and FC data.

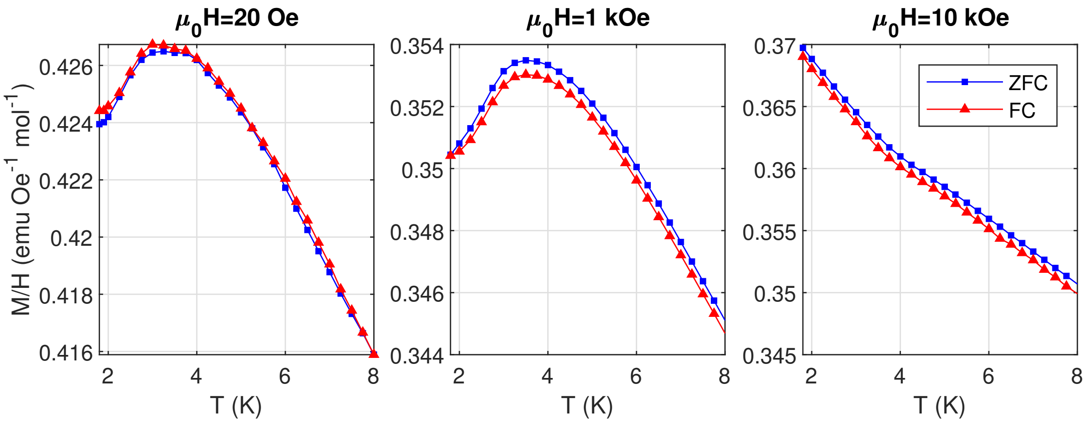

Figure S9: ZFC versus FC magnetic susceptibility χ of Ba_2TbSbO_6 under applied fields of 20, 1,000, and 10,000 Oe. Error bars (from ± 0.1 mg mass error) are smaller than data points.

Table S5: Irreversible temperature T_{irr} at which the ZFC and FC magnetic susceptibility χ of Ba_2TbSbO_6 diverge for different applied fields $\mu_0 H$.

$\mu_0 H$ (Oe)	T_{irr} (K)
20	7
1,000	> 30
10,000	100

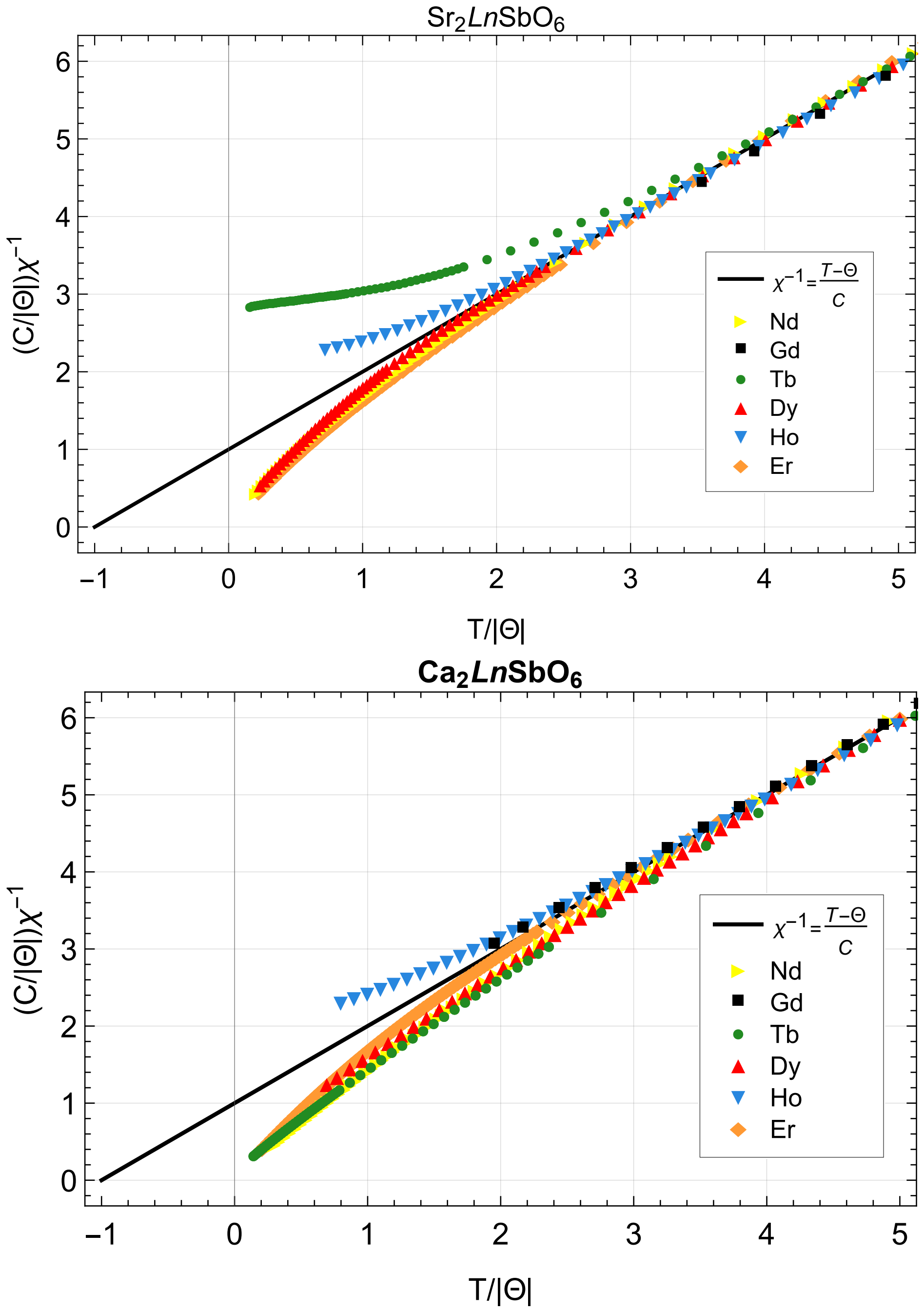

Figure S10: Inverse magnetic susceptibility χ^{-1} of $A_2Ln\mathrm{SbO}_6$, A ={Ba, Sr, Ca}. Both axes are scaled to be dimensionless using the appropriate factors of the Curie constant C and Curie temperature Θ from the Curie-Weiss fit for each material. The predicted dimensionless susceptibility for paramagnetic (uncoupled) spins is shown by the black line, $(C/|\Theta|)\chi^{-1} = T/|\Theta| + 1$. Positive (negative) deviations from this line indicate values of the magnetic susceptibility that are smaller (greater) than that expected for uncoupled spins, which can be an indication of antiferromagnetic (ferromagnetic) short-range correlations above the ordering temperature.[10,11]

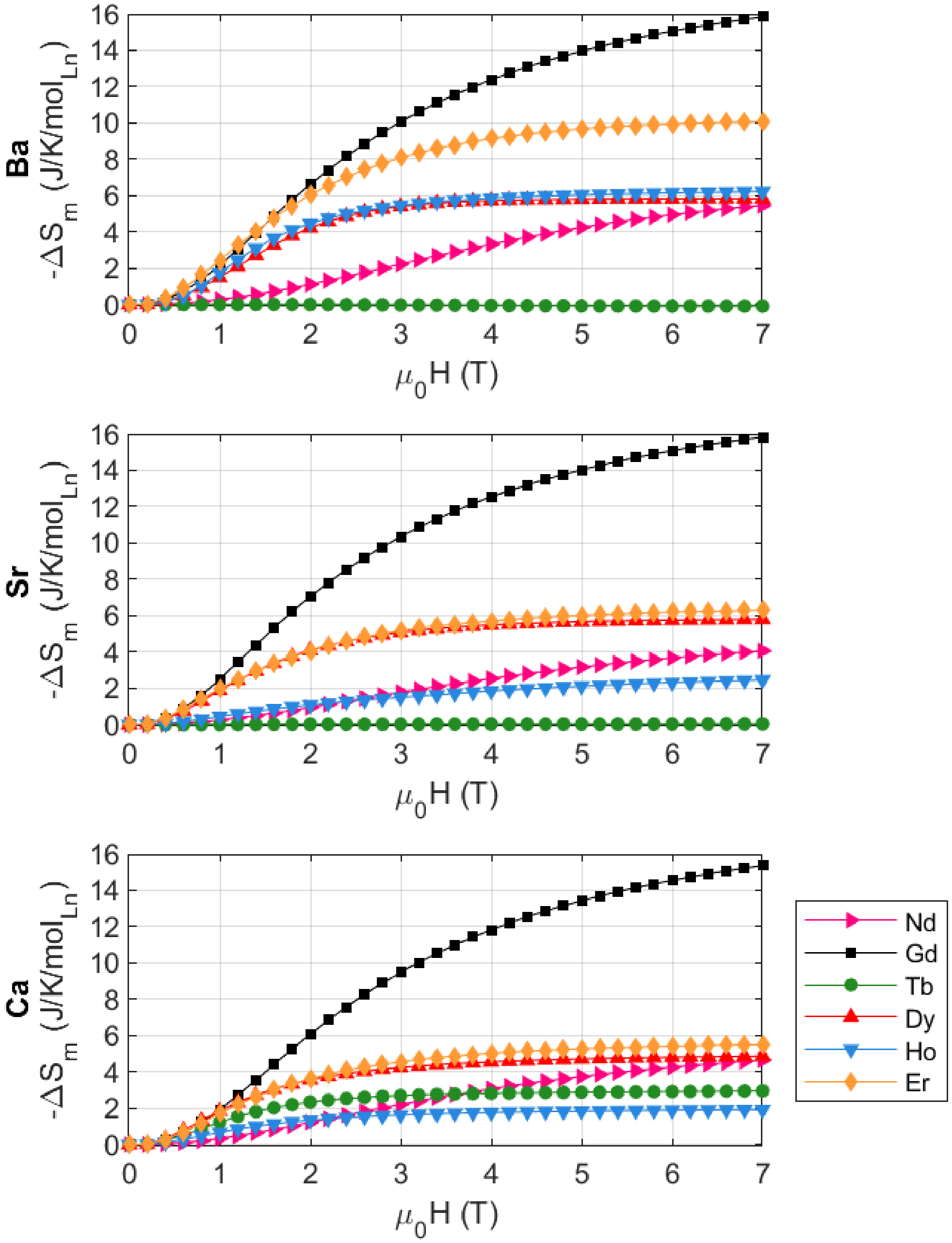

Figure S11: Isothermal magnetic entropy change ΔS_m at 2 K as a function of maximum applied magnetic field $\mu_0 H$ for $A_2 Ln\mathrm{SbO}_6$ where $A = \{\mathrm{Ba}^{2+}, \mathrm{Sr}^{2+}, \mathrm{Ca}^{2+}\}$ and $Ln = \{\mathrm{Nd}^{3+}$-$\mathrm{Er}^{3+}\}$. Solid lines are shown to aid the eye.

References

(1) Fennell, T.; Bramwell, S.; Green, M. Structural and magnetic characterization of Ho_3SbO_7 and Dy_3SbO_7. *Canadian Journal of Physics* **2001**, *79*, 1415–1419.

(2) Hinatsu, Y.; Doi, Y. High-temperature X-ray diffraction measurements of fluorite-related rare earth antimonates Ln_3SbO_7 (Ln=Nd, Tb) and their magnetic properties. *Journal of Solid State Chemistry* **2014**, *217*, 16–21.

(3) Hinatsu, Y.; Ebisawa, H.; Doi, Y. Magnetic properties of orthorhombic fluorite-related oxides Ln_3SbO_7 (Ln=rare earths). *Journal of Solid State Chemistry* **2009**, *182*, 1694–1699.

(4) Koskelo, E. C.; Liu, C.; Mukherjee, P.; Kelly, N. D.; Dutton, S. E. Free-Spin Dominated Magnetocaloric Effect in Dense Gd^3+ Double Perovskites. *Chemistry of Materials* **2022**, *34*, 3440–3450.

(5) Blundell, S. *Magnetism in Condensed Matter*; Oxford Master Series in Condensed Matter Physics 4; Oxford University Press, 2001.

(6) Calder, S. et al. Magnetic properties of Ba_2HoSbO_6 with a frustrated lattice geometry. *Phys. Rev. B* **2010**, *81*, 064425.

(7) Mukherjee, P. Investigation of the magnetic and magnetocaloric properties of complex lanthanide oxides. Ph.D. thesis, University of Cambridge, Cambridge, Cambridgeshire, UK, 2018; Publisher: Apollo - University of Cambridge Repository.

(8) Sackville Hamilton, A.; Lampronti, G.; Rowley, S.; Dutton, S. Enhancement of the magnetocaloric effect driven by changes in the crystal structure of Al-doped GGG, $Gd_3Ga_{5\text{-}x}Al_xO_{12}$ ($0 \leq x \leq 5$). *Journal of Physics: Condensed Matter* **2014**, *26*, 116001.

(9) Dun, Z. L.; Trinh, J.; Lee, M.; Choi, E. S.; Li, K.; Hu, Y. F.; Wang, Y. X.; Blanc, N.; Ramirez, A. P.; Zhou, H. D. Structural and magnetic properties of two branches of the

tripod-kagome-lattice family $A_2R_3Sb_3O_{14}$ (A = Mg, Zn; R = Pr, Nd, Gd, Tb, Dy, Ho, Er, Yb). *Phys. Rev. B* **2017**, *95*, 104439.

(10) Melot, B.; Drewes, J.; Seshadri, R.; Stoudenmire, E.; Ramirez, A. Magnetic phase evolution in the spinel compounds $Zn_{1\text{-}x}Co_xCr_2O_4$. *Journal of Physics: Condensed Matter* **2009**, *21*, 216007.

(11) Dutton, S. E.; Huang, Q.; Tchernyshyov, O.; Broholm, C. L.; Cava, R. J. Sensitivity of the magnetic properties of the $ZnCr_2O_4$ and $MgCr_2O_4$ spinels to nonstoichiometry. *Phys. Rev. B* **2011**, *83*, 064407.
